# Measuring Local Exothermic Effects During the Oxidative Coupling of Methane Using *Operando* Luminescence Thermometry

**DOI:** 10.1002/anie.1717647

**Published:** 2026-06-30

**Authors:** Daniël W. Groefsema, Freddy T. Rabouw, D. Michiel Boele, A.N. René Bos, Alexander P. van Bavel, Bert M. Weckhuysen

**Affiliations:** ^1^ Inorganic Chemistry and Catalysis Group Institute for Sustainable and Circular Chemistry Department of Chemistry Faculty of Science Utrecht University Utrecht The Netherlands; ^2^ Soft Condensed Matter and Biophysics Group Debye Institute for Nanomaterials Science Faculty of Science Utrecht University Utrecht The Netherlands; ^3^ Shell Global Solutions International B.V. Amsterdam The Netherlands; ^4^ Laboratory for Chemical Technology Ghent University Ghent Belgium

**Keywords:** heat transfer, high‐temperature chemistry, luminescence, methane activation, *operando* spectroscopy

## Abstract

Oxidative coupling of methane (OCM) into ethylene is a very promising process for the valorization of methane. However, the high operating temperatures, severe exothermicity, and poor selectivity at high conversion rates have hindered its industrialization. Measurements of local catalyst temperatures, let alone research into the determining experimental parameters, are still scarce. Here, we use *operando* luminescence thermometry (LT) to measure local catalyst temperatures during the OCM reaction. This analytical technique reveals catalyst temperatures exceeding the oven temperature under inert conditions by almost 250°C. We observe a dependence of the local catalyst temperature on the amount of heat generated by the OCM process, and we show that the heat transfer and resulting catalyst temperature in the reactor are strongly influenced by experimental conditions and reactor/catalyst dimensions. This work showcases the opportunities of LT to measure catalyst temperatures and get more insight into high‐temperature catalytic processes and their heat management, leading to crucial insights for the further optimization of chemical processes, such as OCM.

## Graphical abstract

*Operando* luminescence thermometry shows that the local catalyst temperature can exceed the set oven temperature by almost 250°C during the exothermic oxidative coupling of methane process. We demonstrate how the reactor dimensions, catalyst grain size, and reactant feed mixture affect the local temperature. These experiments provide important insights for optimizing the reactor and catalyst design and showcase the use of *operando* luminescence thermometry.

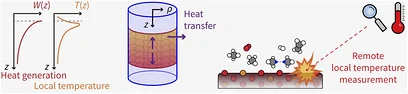

## 1 Introduction

Methane, the main component of natural gas, is widely considered a cheap and abundant carbon feedstock [1]. Natural gas is often recovered as a byproduct of oil extraction at remote locations [2]. Since gas transport is inefficient and economically unattractive, this ‘stranded’ methane is often flared as waste, releasing CO_2_ gas into the atmosphere. In addition to fossil methane sources, we expect an increasing amount of biomethane becoming available, originating, for example, from anaerobic wastewater treatment processes [3]. Thus, methane utilization is of increasing importance in view of circulating waste streams. Methane conversion processes are, however, often costly and complex because of the large activation energy of the C–H bond and the higher reactivity of the related reaction products (e.g., methanol and ethylene) relative to the reactant. The development of more efficient technologies for the conversion of methane into valuable chemicals is therefore of great interest. Since the first reports in the 1980s [4, 5, 6], oxidative coupling of methane (OCM) into ethylene has attracted a lot of attention as a potential methane valorization method. Direct conversion of methane can eliminate costly intermediate conversion processes in the production of value‐added chemicals, and ethylene is one of the most widely used carbon building blocks in the (petro)chemical industry [7]. However, despite decades of research, viable industrialization of the OCM technology has not yet been achieved [8].

Local temperatures and exothermicity play a crucial part in the performance of OCM catalyst materials and related chemical reactors. Temperatures between 600 and 1000°C are typically required to activate the C–H bond in methane for most of the reported OCM catalyst materials [9, 10, 11]. Many of the chemical reactions known to take place in the OCM process are exothermic, which can lead to differences between the set oven temperature and the actual catalyst temperature in lab‐scale experiments, hot spot formation in the catalyst bed, or even thermal runaway [12, 13, 14]. Different studies under various conditions have shown axial temperature gradients and hot spots in the catalyst bed exceeding the average reactor oven temperature, in some cases by more than 300°C, which can severely influence the catalyst stability and performance. These measurements are often performed with thermocouples inside the catalyst bed using quartz capillary wells [13, 14]. The main disadvantages in these temperature measurements are that the thermocouple does not measure the catalyst temperature directly, it can affect the heat and gas flow in the reactor, puts restrictions on the reactor design, and the position of the measurement is not always well defined. Examples of circumventing these challenges have been reported, most notably using infrared (IR) thermometry. In this way, temperature gradients have been observed in OCM, as well as concentration gradients and correlation with exothermic reactions affecting the selectivity to C_2_‐products [15, 16].

Luminescence thermometry (LT), based on temperature‐dependent emission characteristics of luminescent lanthanide ions, has proven to be a valuable tool for remote temperature measurements in various sample environments, including catalytic reactions at temperatures up to 600°C [17, 18, 19, 20, 21, 22]. By analyzing temperature‐induced changes in relative emission intensities, the temperature of the emitting material can be determined via the temperature‐dependent Boltzmann distribution model (see Section 1 of the Supporting Information for more details) [23, 24]:

(1) $$I_{2}/I_{1}=Ce^{-\Delta E/k_{B}T}$$

where, *I*_1_ and *I*_2_ are the relevant emission intensities, *C* is a constant pre‐factor, Δ*E* is the energy gap between the emitting energy levels, *k*_B_ is the Boltzmann constant, and *T* is the temperature. One of the advantages of this technique, is that it is essentially self‐referencing, as opposed to, for example, IR thermometry techniques, which may be affected by absorption or scattering in the optical path [24]. Furthermore, the LT material can be incorporated in the catalyst material, leading to an inherent measurement of the actual catalyst temperature. In principle, the spatial resolution of different optical techniques may be comparable, because they are theoretically diffraction‐limited. Qualitative and quantitative comparison, however, will depend on various instrumentation and experiment design characteristics. Depending on the reactor setup available, LT may provide a relatively easy to implement analysis technique, complementing the earlier reported remote sensing approaches for *operando* catalyst monitoring.

An overview of previously reported LT applications is given in Section 2, Supporting Information. Although valuable insights have been obtained in the first applications of LT in a reactive environment, in‐depth LT studies of the effect of experimental parameters on the resulting catalyst temperature and relevant heat generation and transport phenomena are still lacking. Unfortunately, the emission intensity of many LT materials decreases at elevated temperatures because of degradation of the host material and thermal quenching of the luminescence via non‐radiative relaxation pathways [25, 26]. Furthermore, many of the reported catalytic LT applications require some form of optical focusing (e.g., microscope objectives or a focused probe) to obtain a sufficient collection efficiency for thermometry at high temperatures. This puts severe limitations on the application of the reported techniques to an *operando* high temperature reactor. Previous reports of LT measurements during catalytic experiments are scarce and typically limited to a maximum temperature of 600°C, as mentioned in reviews and perspectives by Brites et al. [27], Gao et al. [28], and Harrington et al. [29]. Due to the requirement of high temperatures for the OCM process, often well above the current limit of 600°C [9, 10, 11], application of LT techniques to study the local temperature of an OCM catalyst requires a host material with high thermal stability and lanthanide dopants with a high thermal quenching temperature. We recently reported the first *operando* application of LT in a working catalytic process above 600°C [30].

**FIGURE 1:**
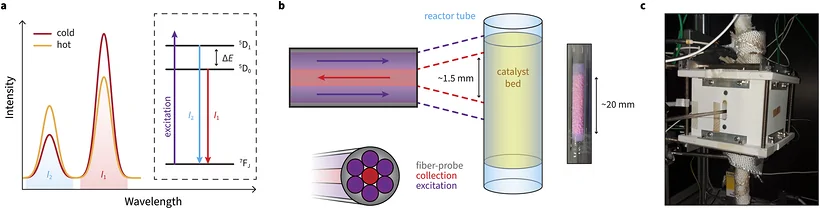
Overview of the *operando* thermometry approach in this work. (a) Schematic illustration of the ratiometric luminescence thermometry (LT) principle. (b) Illustration of the optical probe used for *operando* catalyst monitoring (not to scale) and a picture of one of the reactor tubes used in this work, showing the luminescence of the Y_2_O_3_:Eu catalyst bed upon UV excitation. (c) Picture of the reactor setup with the optical probe inserted into the oven.

In this work, we demonstrate how the local temperature in an operational OCM reactor can be tuned by the catalyst, reactor design, their interplay, and the reactant feed composition and flow rate. We use Y_2_O_3_ as a host material with low phonon energy, and Eu^3+^ as a lanthanide with a large energy gap between the emitting energy levels and the lower‐lying levels, to enable LT at OCM temperatures. We take advantage of the fact that Y_2_O_3_ is also able to activate methane for OCM, albeit moderately [31, 32]. Thereby, we use Y_2_O_3_:Eu as a hybrid catalyst and thermometry material for *operando* studies of local temperatures during the OCM process. The local temperature is determined from the emission spectrum due to the thermally coupled ^5^D_0_ and ^5^D_1_ energy levels of Eu^3+^, as schematically illustrated in Figure 1a. Because the optical thermometry signal is emitted from within the catalyst material, this analytical technique inherently measures the temperature of the catalyst material during the reaction. In this approach, we do not attempt to uncover catalyst‐specific behavior or details. Instead, we focus on the local catalyst temperature and thermal management facets in our experiments and subsequent modeling, using the Y_2_O_3_:Eu catalyst and thermometer as an experimental tool.

We use an in‐house designed reactor oven fitted with a slit in the oven to insert an optical fiber probe. A 392 nm laser is coupled into the optical fiber and transmitted through six excitation fibers toward the Y_2_O_3_:Eu catalyst to excite the Eu^3+^ ions. The resulting emission signal is collected by a read fiber in the same probe and transmitted toward the detector to measure emission spectra. This is schematically illustrated in Figure 1b. Based on the numerical aperture (NA) of the optical fibers, and on manually moving the probe along the catalyst bed, we estimate an upper limit to the axial spot size in our measurements of ∼ 1.5 mm (more details in Section 3, Supporting Information). Although this spatial resolution is not optimal, it is small enough to allow for sufficiently local measurements of temperature gradients in our experiments. A photograph of one of the reactor tubes used in this work is shown on the right in Figure 1b. In this photograph, the catalyst is illuminated with a simple handheld UV lamp, showing a reddish hue caused by the luminescence of Eu^3+^ in the catalyst material. A picture of the reactor setup is shown in Figure 1c. More details of the setup and the experimental methods can be found in Section 4, Supporting Information.

Ethylene formation over the Y_2_O_3_:Eu catalyst in our reactor system is confirmed by gas chromatography (GC) analysis of the products. By varying the reactant gas flow rate and composition, the steady‐state heat generation rate in the reactor can be varied while monitoring the local temperature. This reveals a roughly linear relation between the heat generation and the observed exothermic temperature increase in the catalyst material, relative to the oven temperature under inert conditions, at constant oven heating. By varying the reactor diameter, catalyst grain size, and reactant feed composition, we identify the catalyst bed dimensions and grain size as dominant parameters determining the local catalyst temperature during the OCM process in our laboratory‐scale reactor. Intuitive explanations for the experimentally observed trends are supported by a simple analytical model that describes the local temperature in terms of catalyst bed dimensions, total heat generation, and oxygen conversion. The model is based on a position‐dependent local heat generation rate, heat dissipation through the catalyst bed, and heat loss to the environment. This approach reveals that the local temperature is governed by the catalyst bed dimensions and catalyst grain size via a balance between the effective thermal conductivity in the catalyst bed and a heat loss coefficient for heat transfer out of the bed.

Our non‐invasive *operando* local temperature measurements and subsequent description of the recorded temperature in terms of observed reaction results, reactor and catalyst dimensions, and heat transfer coefficients, show the applicability of LT to measure, describe and model local temperature and reaction profiles in an operational OCM reactor. For the first time, we consider reaction thermodynamics and vary the reactor design, and we observe and model the resulting changes in temperature. We provide a new experiment‐based framework of thinking concerning experimental studies and modeling of the parameters that govern the local temperature and, hence, local reaction kinetics, and we show the possibility of applying this approach in both steady‐state and transient conditions.

## 2 Results and Discussion

### 2.1 Activity for the Oxidative Coupling of Methane and Temperature‐Dependent Emission of Y_2_O_3_:Eu

**FIGURE 2:**
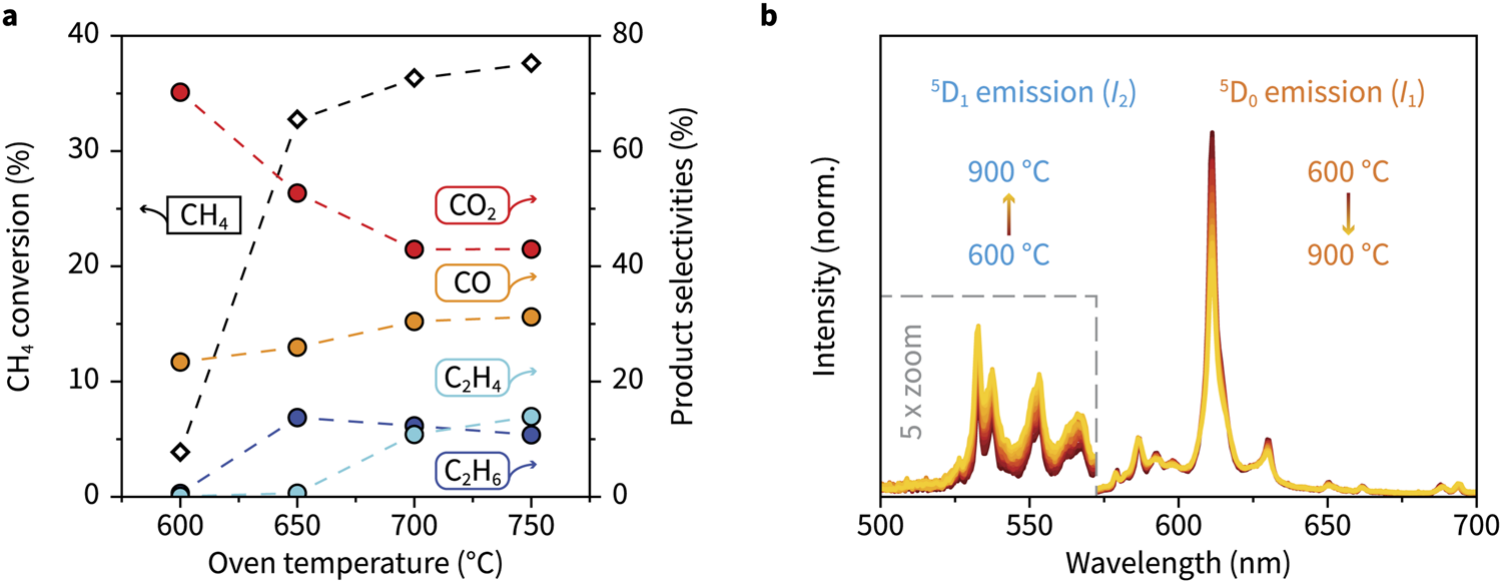
Catalytic performance in the oxidative coupling of methane (OCM) process and temperature‐dependent emission for the Y_2_O_3_:Eu (4 at%) catalyst/thermometry material. (a) Methane conversion (left axis, open symbols) and selectivities toward C_2_ and CO_x_ products (right axis, colored symbols) as a function of the oven temperature. Dashed lines are guides to the eye. (b) Normalized emission spectra of the temperature‐dependent europium emission from Y_2_O_3_:Eu, recorded ex situ at temperatures ranging from 600°C (red) to 900°C (yellow) after *λ*_ex_  =  392 nm excitation. Background spectra were recorded and subtracted for all spectra.

Figure 2a shows the methane conversion, selectivity toward ethane and ethylene (C_2_), and CO_x_ products for the Y_2_O_3_:Eu catalyst material, as a function of the set oven temperature. Most importantly, carbon–carbon coupling into ethane and ethylene is indeed observed for this catalyst in our reactor. The methane conversion and C_2_ selectivities increase with increasing oven temperature in the range of 600–750°C. Additionally, the ethylene/ethane ratio increases at higher temperatures, corresponding to increased dehydrogenation of the primary ethane products in OCM. The sharp increase in conversion between 600 and 650°C can be attributed to operation around the light‐off temperature of the Y_2_O_3_:Eu catalyst. The observed trends agree well with reported literature for various OCM catalysts, confirming that our experimental setup is suitable for moderate OCM performance [32]. The calculations for conversion and selectivity are described in Section 5, Supporting Information.

Figure 2b shows the emission spectra for the Y_2_O_3_:Eu catalyst material recorded ex situ (in a small heating stage under ambient air) at different temperatures in the OCM temperature range of 600–900°C. The emission lines at wavelengths shorter than 575 nm originate from the ^5^D_1_ energy level of Eu^3+^, while the emission lines at longer wavelengths are mostly from the ^5^D_0_ level. A temperature‐dependent increase of the ratio between emission intensities from the ^5^D_1_ and ^5^D_0_ energy levels is observed. The luminescence intensity ratio between emission from these levels can thus be used for remote temperature sensing.

### 2.2 In Situ Calibration and Processing of Temperature‐Dependent Europium Emission

**FIGURE 3:**
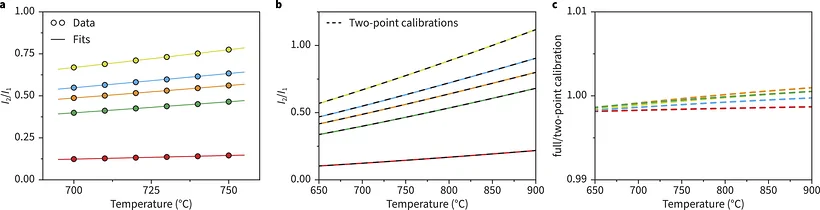
In situ calibration measurements of the temperature‐dependent europium emission. (a) Luminescence intensity ratio (LIR) as a function of temperature between 700–750°C, for varying integration ranges (indicated with colors). Circles indicate the measured LIR, solid lines show the fit of Equation (1) to the data, with fitted values of Δ*E* = 2250 cm^−1^ and *C* = 3.44 (red); 1968 cm^−1^ and 8.95 (orange); 2038 cm^−1^ and 13.61 (yellow); 2119 cm^−1^ and 9.16 (green); and 1985 cm^−1^ and 10.32 (blue). (b) Calibration curves from panel a (colored lines) and corresponding two‐point calibration curves fitted to the LIR at temperatures of 700 and 750°C (black, dashed lines) as a function of temperature between 650–900°C, with fitted values for the corresponding two‐point calibrations of Δ*E* = 2248 cm^−1^ and *C* = 3.44; 1961 cm^−1^ and 8.86; 2031 cm^−1^ and 13.50; 2113 cm^−1^ and 9.10; and 1980 cm^−1^ and 10.27. (c) Full calibration curves divided by the two‐point calibration curves as a function of temperature between 650–900°C.

For measurements in the reactor, scattering and absorption processes and refractions on the curved quartz surface can distort the recorded emission spectra, depending on the exact alignment of the optical probe. The luminescence intensity ratio (LIR), and thus the temperature read‐out, is consequently distorted by a temperature‐independent factor, which must be corrected for with a dedicated in situ calibration for each experiment. We expect that systematic errors are significant for the *operando* temperature measurements and that analyses with different integration ranges will be affected differently by systematic errors. To estimate the magnitude of the systematic uncertainty, we perform multiple slightly different analysis procedures. The analysis procedures differ by the wavelength ranges used to integrate the ^5^D_1_ and ^5^D_0_ emissions (see Section 6, Supporting Information). The recorded temperatures reported in this study are the average of the read‐out temperatures from the analyses with different integration ranges. We take the smallest and largest value of *T*_read‐out_ from the analyses with different integration ranges as an estimate for the magnitude of the systematic uncertainty. Figure 3a shows calibration measurements recorded in situ at six different temperatures between 700 and 750°C under static N_2_ atmosphere, for the different integration ranges. The calibration curves were fitted to the temperature‐dependent intensity ratio of Equation (1) based on the Boltzmann distribution (Figure 3a). The fitted energy difference between the emitting levels depends on the analysis procedure, ranging between Δ*E* *=* 1968 cm^−1^ and Δ*E* *=* 2250 cm^−1^. These values are slightly larger than previously reported [33], but we conclude that the temperature dependence of the LIR is nevertheless described well with the Boltzmann distribution model in the OCM temperature range.

Because the calibrations are time‐consuming, we examine the possibility of using a two‐point in situ calibration for subsequent experiments. We performed two‐point calibration using only the data points at 700 and 750°C. Figure 3b shows the full calibration curves from Figure 3a (colored lines), along with the corresponding calibration curves obtained from the two‐point fits (black, dashed lines overlaid). Both are extrapolated over a wide temperature range of 650–900°C, but the good agreement between the full and two‐point calibrations is maintained. Figure 3c quantifies the difference between the two‐point and full calibration curves, in terms of the relative LIR of the two calibrations. We find an excellent match between the full calibration curves and the two‐point calibrations, comfortably within ±0.5% over the whole temperature range. We check the effect of the small deviations between the full and two‐point calibrations on the read‐out temperature by inserting the LIR value of the full calibration curves at temperatures of 650 and 900°C into the corresponding two‐point calibration curve, to obtain a two‐point calibrated temperature‐read out from the inserted LIR values. The resulting temperatures from the two‐point calibrations are off by a maximum of only 0.55°C at 900°C, compared to the full calibrations. This means that the use of two‐point calibrations is justified for following experiments.

### 2.3 Steady‐State Catalyst Temperature During the Oxidative Coupling of Methane

**FIGURE 4:**
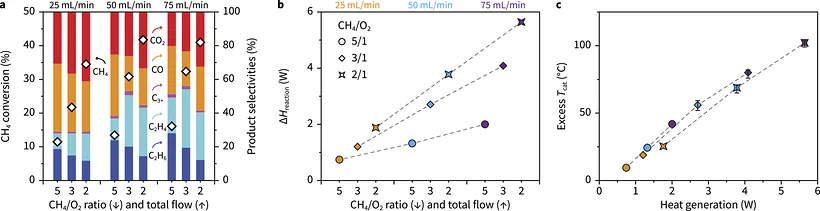
Catalytic performance, heat generation, and local excess catalyst temperature (defined as the difference between the measured catalyst temperature and the oven temperature under inert conditions) for Y_2_O_3_:Eu during the oxidative coupling of methane (OCM) process under various reactant flows. (a) Methane conversion (left axis, open symbols) and selectivities toward C_2_, C_3+,_ and CO_x_ products (right axis, colored bars) for different total flow rates (top axis) and CH_4_/O_2_ ratios (bottom axis). (b) Calculated enthalpies of reaction, based on in‐line gas chromatography (GC) product analysis and the standard enthalpies of formation of reactants and products, for the conversions and product distributions shown in panel a. (c) Local excess catalyst temperature, relative to the oven temperature under N_2_ (*T*_oven_ = 672°C) in the top of the catalyst bed as a function of the total heat generation. Colors represent the different total flow rates and symbols indicate the different CH_4_/O_2_ ratios, as in panel b. Data points corresponding to identical CH_4_/O_2_ ratio are connected with dashed lines in panels b and c. The error bars indicate the estimated systematic uncertainty. Error bars smaller than the marker size are omitted for clarity (∼ 5°C). Random errors are <1°C for all data.

To gain insight in the relation between the steady‐state heat generation rate and the catalyst temperature, first the total reactant flow and the CH_4_/O_2_ ratio were varied to tune the exothermicity of the overall reaction, at constant oven heating. Figure 4a shows the OCM activity for the varying flow conditions of CH_4_/O_2_ ratio 2–5 and total flow rate 25–75 mL min^−1^, corresponding to gas hourly space velocities (GHSV) of about 15,000–45,000 h^−1^ (dividing the total volumetric flow rate by the volume of the catalyst). Here, different methane conversions and product distributions are observed, depending on the applied reactant flows. An increase in selectivity toward ethylene is observed for increasing total flow and increasing O_2_ concentration. Notably, the CH_4_ conversion often increases with increasing flow rate, for example going from 25 to 50 mL/min for all CH_4_/O_2_ ratios. Although this behavior contradicts some generally observed trends in OCM, it has been observed before, both computationally and experimentally. For example, Vanderwalle et al. have modelled an OCM lumped thermal reactor with a Sr/La_2_O_3_ catalyst (with very detailed kinetics), where the trend of simultaneously increasing methane conversion and selectivity toward C_2_‐products was observed, in this case as a function of inlet temperature [34]. Additionally, Dedov et al. have screened various rare earth oxide catalyst, where this behavior is observed for some reaction conditions over an Y_2_O_3_ catalyst among others [31], and Wu et al. have reported a simultaneously increasing methane conversion and selectivity toward C_2+_‐products with increasing reactor temperature over a Gd_2_O_3_ catalyst [35].

We believe, in our case, the observed increase of CH_4_ conversion and selectivity toward C_2+_‐products may be an effect of an increasing catalyst temperature with increasing flow rate. Simultaneously, yet separately, the increasing selectivity toward C_2_‐products may be due to the higher temperatures achieved at higher flow rates. This is in contrast with the possible phenomenon of decreasing selectivity toward C_2_‐products because of overoxidation, due to increasing conversion by increasing residence time in an isothermal reactor.

The corresponding heat generation for the experiments shown in Figure 4 was calculated as the difference in standard enthalpy of formation of the reactant and product mixtures, shown in Figure 4b (see Section 7, Supporting Information for more details). A clear dependence of heat generation on the applied reactant flow and composition is observed, confirming the possibility to tune the exothermicity via the reactant feed.

To find a relation between the steady‐state heat generation rate in the catalyst bed and the temperature of the catalyst material, local catalyst temperatures were measured in the top of the catalyst bed using *operando* LT, for varying exothermicity via the flow conditions as described above. Figure 4c shows the measured local excess catalyst temperature, defined as the difference between the constant oven temperature under inert conditions (*T*_oven_ = 672°C in the experiments in Figure 4) and the measured catalyst temperature, as a function of the heat generation rate as derived from the GC analysis. The in situ two‐point calibrations shown in Figure 3 were used for the temperature measurements shown in Figure 4c. The error bars indicate the estimated systematic uncertainty, while random errors due to noise are much smaller (estimated 0.13–0.57°C). A roughly linear trend of increasing excess catalyst temperature with the heat generation rate is observed, which appears independent of the total flow and CH_4_/O_2_ ratio. An exothermic temperature increase is already visible for the smallest amount of exothermic heat release in the studied conditions, with a heat generation rate <1 W and excess *T*_cat_ = 9°C. In the most exothermic conditions, with a heat generation of 5.6 W, the excess *T*_cat_ reaches over 100°C.

### 2.4 Experimental Parameters Governing the Local Temperature

**FIGURE 5:**
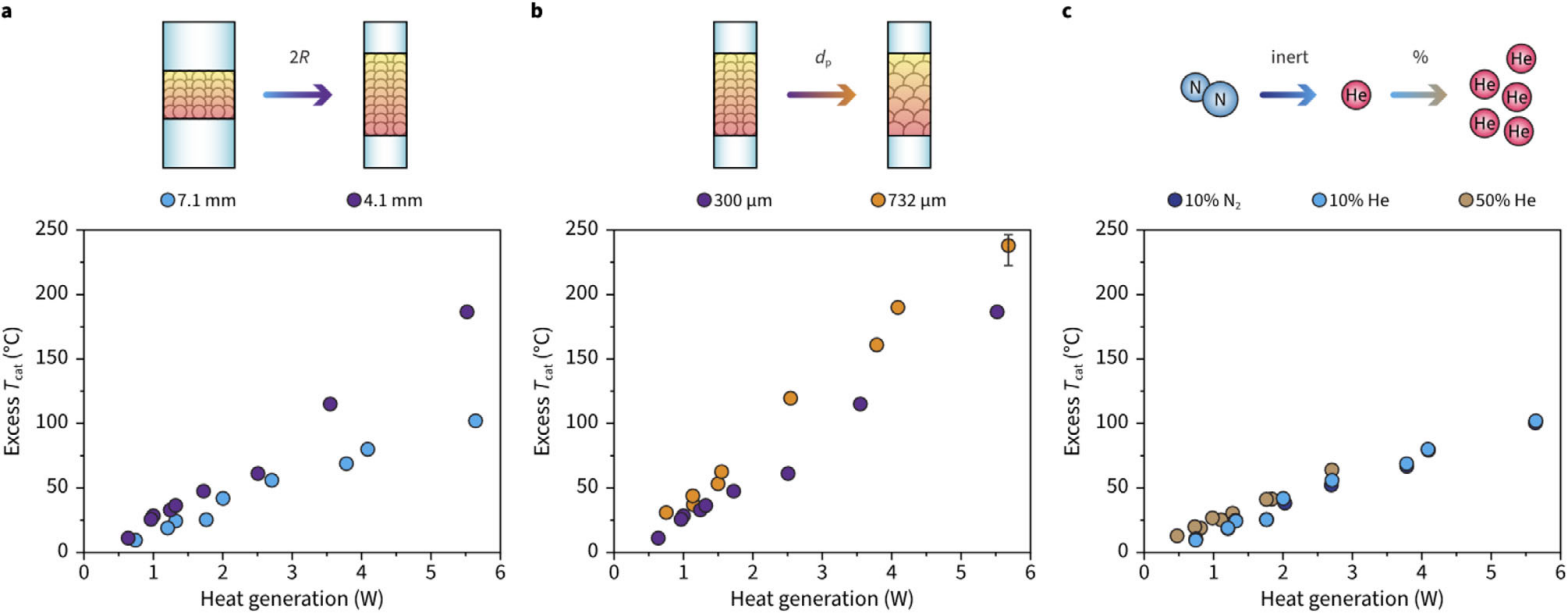
Local excess catalyst temperature in the top of the catalyst bed, relative to the oven temperature under inert conditions, during the oxidative coupling of methane (OCM) process, as a function of the total heat generation under various reactant flows. (a) In reactor tubes with 2*R* = 7.1 and 4.1 mm (*d*_p_ = 300 µm). (b) For catalyst grain sizes of *d*_p_ = 300 and 732 µm (2*R* = 4.1 mm). (c) For 10% N_2_, 10% He, and 50% He inert diluent in the reactant feed mixture (*d*_p_ = 300 µm, 2*R* = 7.1 mm for 10% inert; and 2*R* = 4.1 mm for 50% inert). Calibration data for these measurements are shown in Section 9, Supporting Information. The total flows and CH_4_/O_2_ ratios are the same as in Figure 4, the indicating colors and symbols have been omitted for clarity. The error bars indicate the estimated systematic uncertainty. Error bars smaller than the marker size are omitted for clarity (∼ 10°C for all panels). Random errors are <1°C for all data.

In addition to the oven temperature and the heat generation due to the reactions that take place, three pathways of heat transport can influence the local catalyst temperature. These are heat transport due to convection via the gas flow, conduction/radiation through the catalyst bed, and heat loss through the reactor wall outwards. In this view, a strategy to study the contributions of varying experimental parameters that affect the heat transport is examined. The variations include the reactor tube diameter 2*R* to vary the surface‐to‐volume ratio of the catalyst bed; the mean catalyst particle grain size *d*_p_ to vary the thermal conductivity through the bed; and the composition and concentration of inert diluent in the reactant feed mixture to induce changes in the convective heat transfer (see Section 8, Supporting Information for schematic illustrations). Figure 5 shows the local excess catalyst temperature as a function of the heat generated by the OCM process for these variations. The oven temperatures without reaction in these experiments were *T*_oven_ = 672°C (2*R* = 7.1 mm, *d*_p_ = 300 µm, 10% He, shown in blue), 657°C (2*R* = 4.1 mm, *d*_p_ = 300 µm, 10% He, shown in purple), 665°C (2*R* = 4.1 mm, *d*_p_ = 732 µm, 10% He, shown in orange), 672°C (2*R* = 7.1 mm, *d*_p_ = 300 µm, 10% N_2_, shown in dark blue), 667°C (2*R* = 4.1 mm, *d*_p_ = 300 µm, 50% He, shown in brown). Random errors are 0.05–0.94°C for all experiments shown in Figure 5. The in situ calibration data for the experiments are shown in Section 9, Supporting Information.

At similar amounts of heat generation, Figure 5a shows a higher excess catalyst temperature (i.e., a steeper slope of *T*_cat_ as a function of heat generation) when the reactor tube diameter is reduced from 7.1 to 4.1 mm, especially in more exothermic conditions (heat generation >3 W). The difference between the two reactor tubes in Figure 5a reaches almost 70°C and the excess local catalyst temperature reaches almost 200°C for the narrow reactor tube (2*R* = 4.1 mm). When the mean catalyst grain size in this reactor is increased from 300 to 732 µm, as shown in Figure 5b, the observed excess catalyst temperature shows an additional increase for similar heat generation rates, up to almost 250°C above the oven set temperature under inert conditions. These observations clearly show that the catalyst bed dimensions strongly influence the local catalyst temperature during the OCM process. Increasing the catalyst grain size has a similar effect, whereas the local excess catalyst temperature is not affected by the composition and concentration of inert diluent under the studied reaction conditions, even at 50% dilution of the reactant feed (shown in Figure 5c). The lower total heat generation at the same gas hourly space velocity for 50% diluent can be ascribed to a lower effective feed rate of reactants. Possibly, pronounced wall channeling of the gas flow may play a role in these observations, potentially promoting unselective gas‐phase oxidation, especially for low tube‐to‐particle diameters (2*R*/*d*_p_). However, we expect this to become important only for very low values of 2*R*/*d*_p_ of well below 10, a typical value generally used as criterion for neglecting wall effects in packed beds, based on, for example, Westerterp et al. [36, 37, 38, 39]. A ratio smaller than 10 is the case only for the larger catalyst particles in the narrow reactor in our experiments. Furthermore, if the larger excess temperature in the slender reactor would be fully attributed to more unselective gas‐phase oxidation, we would expect to see this reflected in the relationship between the heat generation and local excess temperature (i.e., a similar or higher temperature along with larger heat generation). As we show in later sections of this work, we identify heat outflow through the top of the catalyst bed as the dominant cause for this unexpected behavior in our experiments. Detailed information about the radial temperature profile in the catalyst bed would be informative to get a better understanding of this behavior.

The observed consistency in local excess catalyst temperature for varying diluent composition and concentration indicates that heat transfer via convection does not play a significant role in our reaction conditions, which is consistent with the earlier observation that the excess catalyst temperature shows a roughly linear correlation with the heat generation rate, independent of total flow and CH_4_/O_2_ ratio (Figure 4c). To further confirm the negligible effect of convective heat transfer in our experiments, we calculated effective thermal conductivities through the catalyst bed, based on Yagi and Kunii [40], Kunii and Smith [41], and Zehner and Schlünder [42], according to expressions by Froment, Bischoff, and De Wilde [43] for heat conduction through a packed catalyst bed (see Section 10, Supporting Information for more details). These calculations show that the convective contribution to the effective thermal conductivity is smaller than 2.5% in all the studied experimental conditions.

The differences in excess temperature upon varying 2*R* and *d*_p_ observed in Figure 5a,b cannot be explained by changes in the effective thermal conductivity. Decreasing the reactor diameter from 7.1 to 4.1 mm results in a virtually unchanged effective thermal conductivity (<1% increase under all conditions) in the calculations mentioned above. Increasing the grain size from 300 to 732 µm yields an increase in calculated thermal conductivity, which in itself would result in a lower local temperature due to more effective heat dissipation, as opposed to the observed higher temperatures in Figure 5b. Clearly, the thermal conductivity through the catalyst bed alone is not able to explain the observed differences that are shown in Figure 5a,b.

### 2.5 Observation of Temperature Difference over the Catalyst Bed

**FIGURE 6:**
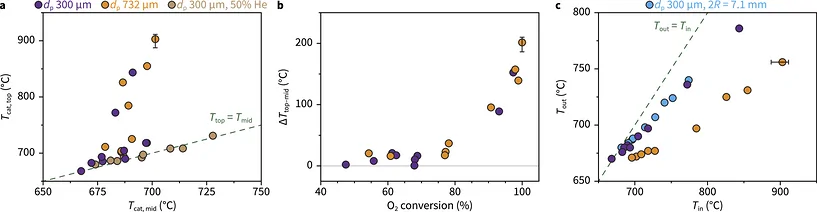
(a) Catalyst temperatures in the top (vertical axis) and middle (horizontal axis) of the 2*R* = 4.1 mm catalyst bed during the oxidative coupling of methane (OCM) process under varying reactant flows, using 10% He diluent for *d*_p_ = 300 (purple) and 732 µm (orange), and 50% He diluent for *d*_p_ = 300 µm (yellow). The dashed green line is a guide to the eye for *T*_top_ = *T*_mid_. (b) The difference between *T*_top_ and *T*_mid_ (Δ*T*_top–mid_) as shown in panel a, as a function of the total oxygen conversion for varying *d*_p_ with 10% He. (c) The local catalyst temperature measured with LT in the top of the catalyst bed (*T*_in_) and the temperature recorded with a thermocouple at the same height on the outside of the reactor tube (*T*_out_), for *d*_p_ = 300 and 732 µm (2*R* = 4.1 mm) and for 2*R* = 7.1 mm (*d*_p_ = 300 µm). The dashed green line is a guide to the eye for *T*_out_ = *T*_in_. The total flows and CH_4_/O_2_ ratios are the same as in the previous experiments. The error bars indicate the estimated systematic uncertainty. Error bars smaller than the marker size are omitted for clarity (∼ 3 and ∼ 12°C for panel a, *x*‐, and *y*‐axis; ∼ 12°C for panel b, *y*‐axis; and ∼ 10°C for panel c, *x*‐axis). Thermocouple read‐outs (panel c, *y*‐axis) are stable over the measurements with a resolution of 1°C. Random errors are <1°C for all data.

For the 2*R* = 4.1 mm reactor, we measured local catalyst temperatures during OCM at two axial positions in the catalyst bed, by aligning optical probes to the top and to the middle of the bed with 9.5 mm spacing between the two probes. This allows us to directly compare differences in local catalyst temperature between the top of the catalyst bed, where hot spots are expected to occur, and the middle of the bed. The placement of a thermocouple fixated to the outside of the reactor tube at the top of the catalyst bed allows us to compare the local catalyst temperature to the temperature on the outside of the reactor wall at the top of the catalyst bed. Figure 6a shows the measured catalyst temperatures in the top and the middle of the catalyst bed for catalyst grain sizes of 300 and 732 µm using 10% He diluent, and for a grain size of 300 µm using 50% He diluent. From these experiments, it becomes clear that a significant temperature gradient over the catalyst bed exists for reactant flows with 10% diluent.

The axial temperature difference in the catalyst bed can reach more than 150°C over an axial distance of 9.5 mm in the catalyst bed for the catalyst grains of *d*_p_ = 300 µm, and more than 200°C for the 732 µm grains. This observation is consistent with strong reaction gradients in the axial direction within the catalyst bed, as is often reported for the OCM process [13, 14, 15]. Considering the oxygen conversion as a measure of reaction gradients (i.e., stronger gradients exist for higher oxygen conversions), we observe a positive correlation between the axial temperature difference Δ*T*_top–mid_ and the oxygen conversion for 10% feed dilution (shown in Figure 6b), especially at the higher oxygen conversions. Upon increasing the diluent concentration to 50%, this difference disappears, and the temperature appears to be equilibrated over the axial measurement positions in the catalyst bed (shown in brown in Figure 6a). At higher activities, using 10% diluent, we observe a difference between the catalyst temperature in the top of the bed (*T*_in_) and the temperature recorded on the outside of the reactor wall at the same height (*T*_out_), as shown in Figure 6c. This difference increases as the local catalyst temperature increases, and more importantly, the difference is independent of the reactor tube diameter, but becomes larger when the catalyst grain size is increased. This indicates that the catalyst grain size affects the local temperature by a change in heat transfer through the reactor wall to the outside.

For indication, we calculate the adiabatic temperature rise for our experiments. For a single reaction, the adiabatic temperature rise (unit°C) is $\Delta T_{\mathrm{ad}}=-\Delta H_{\mathrm{rxn}}^{0}\;x_{\lim}\;X/C_{p}$, with $-\Delta H_{\mathrm{rxn}}^{0}$ the reaction enthalpy (J/mol), *x*_lim_ the mole fraction of the limiting reactant, *X* the conversion, and *C_p_* the heat capacity of the mixture (J/mol K). We base our calculation of the adiabatic temperature rise in our experiments on the total heat generation (horizontal axis in Figures 4c and 5) and the heat capacity of the product mixture: $\Delta T_{\mathrm{ad}}=Q/\sum_{i}n_{i}C_{p,i}$, where *Q* is the total heat of reaction (unit W, see Section 7, Supporting Information for more details), and $\sum_{i}n_{i}C_{p,i}$ is the sum of the molar flow *n_i_* times the heat capacity at constant pressure *C*_*p*,*i*_ for all components *i* in the product mixture. Generally, we calculate values for the adiabatic temperature rise Δ*T*_ad_ ranging between roughly 250–2000°C for all experiments, and the measured catalyst temperature rise Δ*T*_cat_ in the top of the catalyst bed is often an order of magnitude smaller than the calculated adiabatic temperature rise. Furthermore, the maximum ratio of observed Δ*T*_cat_/Δ*T*_ad_ increases from 0.08 to 0.24 when the reactor tube diameter is decreased from 7.1 to 4.1 mm. Stronger thermal effects may be expected in larger reactors.

In the following sections, we will focus on a description of the local catalyst temperature and trends as observed in Figures 5 and 6. In‐depth description of the OCM activity is beyond the scope of this work. For completeness, the results of the GC analysis of the product mixtures, in terms of conversions and selectivities toward C_2+_ and CO_x_ products, are shown in Section 11, Supporting Information.

### 2.6 Modeling of the Gradient Over the Catalyst Bed With Local Temperature, Thermal Conduction, and Heat Loss Rate

Based on the observations with 10% He diluent in Figures 5 and 6, we consider a model describing the recorded local catalyst temperature in terms of the catalyst bed dimensions (radius *R* and length *L*) and the observed total reaction results (heat generation rate *Q* and oxygen conversion $X_{O_{2}}$), considering heat transfer through the catalyst bed with a thermal conductivity *k* and heat loss to the environment with a heat transfer coefficient *α*. A more detailed explanation of our model and assumptions can be found in Section 12, Supporting Information.

Concisely, we consider that the exothermic reactions taking place during the OCM process generate heat at a position‐dependent rate per unit of volume *W*(*r*, *z*) (in cylindrical coordinates). This creates a temperature profile *T*(*r*, *z*) in the catalyst bed. Heat can diffuse through the catalyst bed from regions of higher to regions of lower temperature with a thermal conductivity *k*, and eventually escapes from the catalyst bed either through the top toward the quartz wool (in the negative *z* direction), through the reactor wall to the oven (in the *r* direction) with a heat transfer coefficient *α*, or possibly through the quartz frit in the bottom of the reactor tube (in the positive *z* direction) with the same coefficient *α*. For this model, we neglect positive feedback of heat generation on the reaction rate, which in reality will affect the final heat generation profile. In the final model, we assume that the heat transfer in the radial direction is sufficiently fast, such that heat transfer through the reactor wall and into the oven becomes the rate‐determining step in heat dissipation. We assume that the heat generation rate is independent of *r*, neglecting any radial flow profile (see Section 12, Supporting Information for the reasoning), and that the heat generation peaks at the top of the catalyst bed (*z*  =  0 mm), where we measured higher local temperatures, after which it decays exponentially with *z*:

(2) $$W\left(z\right)=W_{0}\;e^{-z/d}$$

Here, *d* is an estimate of the length scale over which the heat generation drops, obtained from the measured oxygen conversion $X_{O_{2}}$ as

(3) $$d=\frac{L}{\ln\left(\frac{1}{1-X_{O_{2}}}\right)}$$

The full model is presented in Section 12, Supporting Information.

**FIGURE 7:**
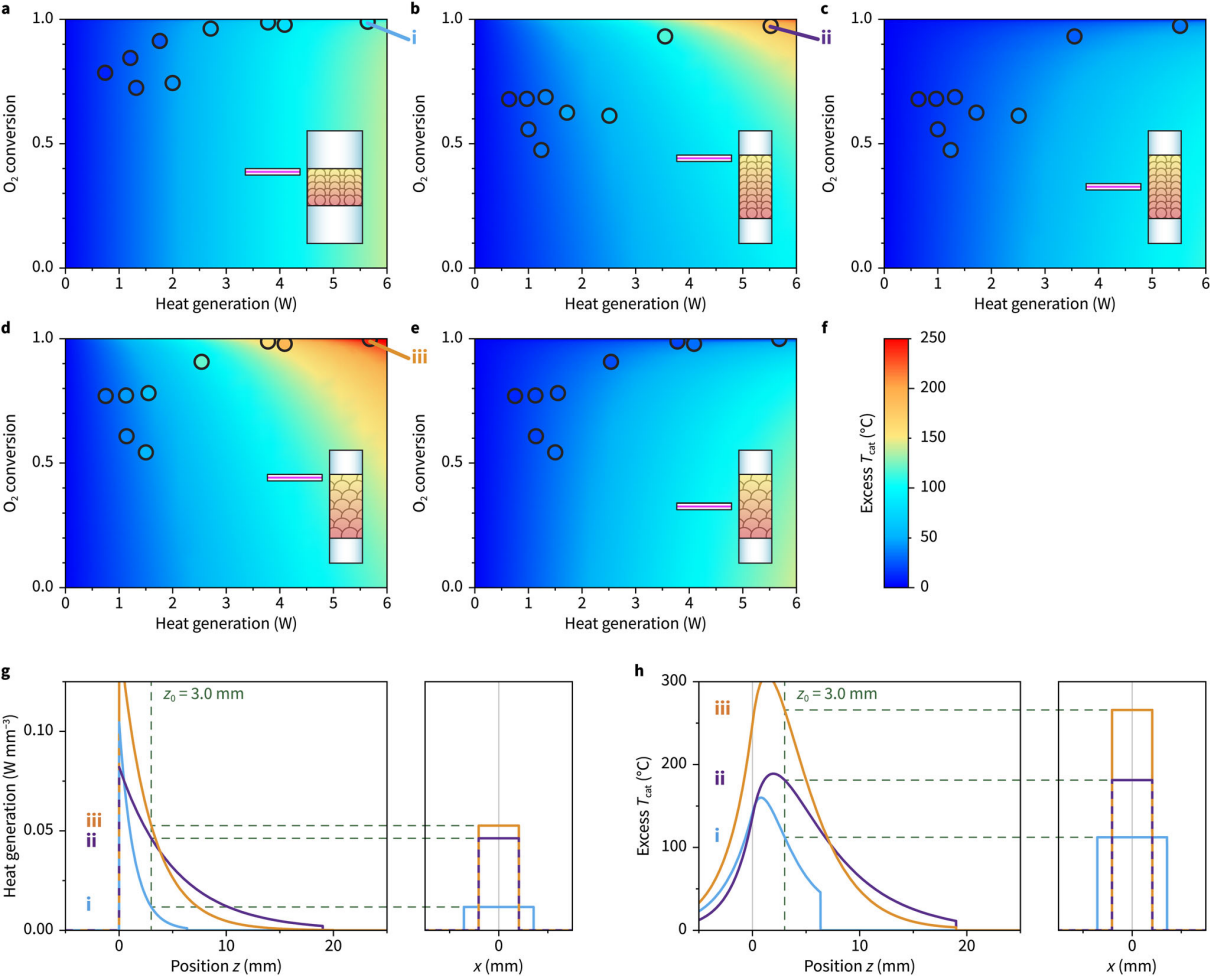
Local excess catalyst temperature as a function of the total heat generation and oxygen conversion during the oxidative coupling of methane (OCM) process. The disks in panels a–e show the experimentally determined heat generation (x axis), oxygen conversion (y axis), and recorded local temperature (color scale) for measurements (a) in the top of the 2*R* = 7.1 mm reactor with *d*_p_ = 300 µm, (b) in the top of the 2*R* = 4.1 mm reactor with *d*_p_ = 300 µm, (c) in the middle of the 2*R* = 4.1 mm reactor with *d*_p_ = 300 µm, (d) in the top of the 2*R* = 4.1 mm reactor with *d*_p_ = 732 µm, and (e) in the middle of the 2*R* = 4.1 mm reactor with *d*_p_ = 732 µm. The total flows and CH_4_/O_2_ ratios are the same as in the previous experiments. The background maps show the results of our model fitted to the experimental data, with fixed *z*_0_ = 3.0 mm (a, b, d) and *z*_0_ = 12.5 mm (c, e); and best‐fit values of *k* = 0.93 W m^−1^ K^−1^ and *α* = 2.2 × 10^2^ W m^−2^ K^−1^ (a, b, c); and *k* = 1.0 W m^−1^ K^−1^ and *α* = 1.7 × 10^2^ W m^−2^ K^−1^ (d, e). The insets show illustrations of the corresponding experimental variations (tube diameter, catalyst grain size, and measurement position). (f) Shared color scale used in panels a–e. (g) Profiles of the heat generation rate *W*(*z*) per unit of volume for selected experiments i–iii in the top right of panels a, b, d. *W*(*z*) was the input for the fit model. The decay along *z* is assumed exponential and determined by experimental values of *Q* and $X_{O_{2}}$. The heat generation profile along *x* (a cross section of the catalyst bed), is uniform by assumption. (h) Profiles of the local excess catalyst temperature *T*(*z*) according to the model fitted to our data. The uniformity along *x* is imposed by the model.

We fit our model (Model 2 from Section 12, Supporting Information) to the experimental data of the experiments with reactor tubes 2*R* = 4.1 and 7.1 mm and catalyst grains *d*_p_ = 300 and 732 µm. Figure 7 shows the experimentally observed local catalyst temperature as a function of the total heat generation and oxygen conversion. Here, different heat generation rates at similar oxygen conversions are caused by differences in product selectivities over the various experiments. The background maps in Figure 7 show the fitting results, fitting for the best‐fit values of *k* and *α* at fixed measurement positions *z*_0_ = 3.0 and 12.5 mm in the top and middle of the catalyst bed, respectively. Since we expect that the thermal conductivity and heat transfer rate are affected by the catalyst grain size, we fit our model separately to the results of the experiments for *d*_p_ = 300 µm (Figure 7a–c) and 732 µm (Figure 7d,e). The model matches the experimental temperatures to within a root‐mean‐square deviation of $\sqrt{\left\langle dT^{2}\right\rangle }$ = 7°C and 14°C for *d*_p_ = 300 and 732 µm, respectively. These numbers are comparable to the maximum estimated systematic measurement uncertainties of 8 and 24°C for the experiments with *d*_p_ = 300 and 732 µm, respectively. A possible cause for deviations between the experimental and modeling results is that the model neglects the increasing importance of radiative heat transfer at higher temperatures. Although the model does not fully match the experimental observations, it reproduces the observed qualitative trends:

- Higher temperature in the narrow reactor (Figures 7b,d) compared to the wider reactor (Figure 7a) with constant *d*_p_
- Lower temperature in the middle (Figures 7c,e) relative to the top (Figures 7b,d) for both values of *d*_p_
- Slightly higher temperature for the larger catalyst grains (Figure 7d) compared to the smaller grains (Figure 7b)

The bottom panels of Figure 7 show the corresponding heat generation profiles (Figure 7g) and temperature profiles (Figure 7h) that follow from our model, for highlighted experiments i, ii, and iii in the top right of Figure 7a,b,d. The modeling and fitting results provide intuitive explanations for the observed trends in our experiments. The temperature at the top of the wider reactor (2*R* = 7.1 mm, experiment i) is lower than at the top of the narrower reactor (2*R* = 4.1 mm, experiment ii) because the heat is dissipated more effectively toward the quartz wool on top of the catalyst bed. Despite the higher peak of heat generation in the 7.1 mm reactor, the temperature peak is lower than in the 4.1 mm reactor. The model allows us to calculate the heat flow across the interface from catalyst to quartz wool, via the heat flow *q*_z_(0) (unit W m^−2^) at *z* = 0: π*R* ^2^ *q*_z_(0) = 1.9 W for experiment i versus π*R* ^2^ *q*_z_(0) = 0.81 W for experiment ii (compare this with the corresponding total heat generation rates of 5.6 and 5.5 W, respectively). Reducing the reactor diameter is typically expected to enhance heat removal for highly exothermic reactions. In our case, next to a reduction of the tube diameter, also the tube bed height is increased, such that the amount of catalyst remains constant. This greatly decreases the cross‐sectional area per bed volume compared to the wall area per bed volume and hence the heat loss from the top to the gas phase. We hypothesize this reduced heat flow upwards out of the top of the catalyst bed in the narrow reactor to be the main cause for the observed higher local temperatures in the top of the bed. These observations may be affected by scale and/or setup‐specific characteristics and are not presented to be a general reactor‐design principle.

The calculated temperature profiles show that the temperature peaks slightly below the top of the catalyst bed due to heat dissipation in the negative *z* direction at the top, where the assumed heat generation rate *W* = 0. This agrees with reported observations of hot spots typically between 0–5 mm below the top of the catalyst bed [13, 14, 15]. The initial heat generation peak (per unit of volume) for experiment iii is even higher than for experiment ii, but decays faster because of the nearly full O_2_ conversion. The temperature difference between experiments ii and iii is more pronounced in the top of the bed, that is, the larger catalyst grains give rise to a larger hot spot formation.

Interestingly, the fitted values for *k* are comparable to those calculated based on the models for heat conduction through a packed catalyst bed based on Froment et al., as mentioned above and in Section 10 of the Supporting Information, even without any input of material properties in our model. Accurate calculation of effective thermal conductivity through a packed catalyst bed is not trivial and depends on i.a. the input value for thermal conductivity *λ*_solid_ of Y_2_O_3_, which varies both in scientific literature and in manufacturer specifications [44, 45, 46, 47]. Taking the smallest and largest reported values, we calculate effective thermal conductivities *λ*_eff_ broadly in the range of 0.2–2.6 W m^−1^ K^−1^ for all our experimental conditions and parameters. The fitted values of *k* = 0.93 and 1.0 W m^−1^ K^−1^ for *d*_p_ = 300 and 732 µm, respectively, are comfortably within this range. The increase of effective thermal conductivity with increasing *d*_p_ is consistent within the calculations and with our fitted values. An increase in effective thermal conductivity of 2–18% is calculated depending on the model and input value for *λ*_solid_. The corresponding relative increase in best‐fit value for *k* of 8% is again comfortably within this range. The fitted heat transfer coefficient *α* decreases from 2.2 × 10^2^ to 1.7 × 10^2^ W m^−2^ K^−1^ for *d*_p_ = 300 and 732 µm, respectively. This indicates that, even though the effective thermal conductivity indeed increases for the larger catalyst grains, reduced heat loss through the reactor wall results in a higher local catalyst temperature in our experimental conditions, which is consistent with our observation of a higher excess catalyst temperature (Figure 5b) and a larger difference between the catalyst temperature and the recorded temperature on the outside of the reactor wall (Figure 6c).

### 2.7 Temperature Response Upon a Change in Heat Generation via the Reactant Feed Composition

**FIGURE 8:**
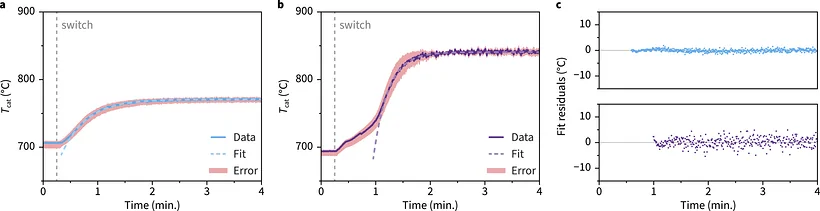
Local catalyst temperature in the top of the catalyst bed as a function of time upon a switch in CH_4_/O_2_ ratio from 5 to 2 in a constant 75 mL min^−1^, 10% He reactant feed for (a) 2*R* = 7.1 mm and (b) 2*R* = 4.1 mm. For both experiments, 0.50 g of Y_2_O_3_:Eu with *d*_p_ = 300 µm was used. Emission spectra were corrected with a background measured at the end of the experiment. The exponential response was fitted to the data (shown in dashed, colored lines), disregarding the first data points up to 40% of the total temperature increase. The reactant switch is indicated in gray, dashed lines. The red band shows the estimated systematic uncertainty. (c) Fit residuals for the fits to the data in panels a and b, for 2*R* = 7.1 mm (top) and 2*R* = 4.1 mm (bottom), respectively.

When the reactant feed composition is changed, the heat generation and local catalyst temperature will change accordingly to reach a new steady state. As explained in Section 13 of the Supporting Information, we expect an exponential response of the transient catalyst temperature over time *T*_cat_(*t*) upon a switch in reactant feed composition, depending on a heat loss rate *k*_loss_:

(4) $$T_{\mathrm{cat}}\left(t\right)=T_{\mathrm{cat},2}+\left(T_{\mathrm{cat},1}-T_{\mathrm{cat},2}\right)e^{-k_{\mathrm{loss}}t}$$

where, *T*_cat,1_ and *T*_cat,2_ are the local steady‐state catalyst temperatures before and after the switch, respectively. Figure 8 shows the excess catalyst temperature as a function of time when the CH_4_/O_2_ ratio in the reactant feed is changed from 5 to 2 in the reactor tubes with 2*R* = 7.1 mm (Figure 8a) and 4.1 mm (Figure 8b), at a constant 75 mL/min total flow with 10% He diluent. The mean catalyst grain size was *d*_p_ = 300 µm for both experiments.

After an initial onset, the exponential temperature response over time is clearly observed in Figure 8. The deviating behavior at shorter times after the switch may be explained by a non‐instantaneous switch in the reactant flow, whereas Equation (4) considers the temperature dynamics for an instantaneous change in gas flow and corresponding heat generation. In reality, the measured response will be affected by i.a. stabilization of the flows via the mass flow controllers and possible mixing of the feed flow in the gas lines before the reactor. We therefore disregard the first datapoints, up to 40% of the total temperature increase, when fitting Equation (4) to the temperature response. The dynamic nature of these experiments poses additional complications in the interpretation because a background correction for each measurement is difficult, and the most suitable measurement and calibration settings vary with local temperature, which varies strongly over these measurements. Nevertheless, here we show the fitting results. The fits are shown in dashed lines in Figure 8 and correspond well with the expected exponential response after the initial onset, yielding fitted values of heat loss rates *k*_loss_ = 0.037 and 0.062 s^−1^ for 2*R* = 7.1 and 4.1 mm, respectively.

As explained in Section 13 of the Supporting Information, we calculate expected heat loss rates based on our model, according to:

(5) $$k_{\mathrm{loss},\mathrm{model}}=\frac{2\alpha }{RC_{p}}$$

For the heat capacity *C*_p_, we use a value of *C*_p_ = 2.9 × 10^−3^ J mm^−3^ K^−1^ for Y_2_O_3_ at 700° C, according to Landa et al. [48]. Using the best‐fit value of *α* = 2.2 × 10^2^ W m^−2^ K^−1^ for *d*_p_ = 300 µm in Equation (5), we calculate expected heat loss rates *k*_loss,model_ of 0.043 and 0.074 s^−1^ for experiments with 2*R* = 7.1 and 4.1 mm, respectively. Compare this with the experimentally observed values of *k*_loss_ = 0.037 and 0.062 s^−1^. Again, our model does not reproduce the experimental observations fully, but the results are close, and the observed trends are consistent between our model and experiments. These results make intuitive sense: The heat loss out of the catalyst bed in the radial direction is proportional to the heat transfer coefficient *α* and inversely proportional to the reactor radius *R*.

## 3 Conclusion

We show that *operando* luminescence thermometry (LT) can quantitatively measure the local catalyst temperature deviating from the set oven temperature because of exothermic reactions during the oxidative coupling of methane (OCM) process in steady‐state and transient operation. In the conditions studied, the local catalyst temperature can reach almost 250°C above the set oven temperature, and differences of over 200°C within the catalyst bed can occur. We observe a linear relation between the generated heat and the temperature increase in the catalyst material, depending on experimental conditions, reactor dimensions and catalyst dimensions. Additional to the heat generation rate during the catalytic reaction, the reactor design and thermal properties and interactions of the reactor‐catalyst system have a strong effect on the local catalyst temperature. The interface between the catalyst bed and the quartz wool layer on top is found to be a determining parameter for heat loss out of the top of the bed. Larger catalyst grains affect the local temperature via a balance of increasing the effective thermal conductivity through the bed and decreasing the heat transfer to the outside. In highly exothermic conditions, the observed excess catalyst temperatures can vary by almost 150°C for similar amounts of heat generation, depending on the reactor and catalyst grain dimensions. Based on our observations, we are able to eliminate convective heat transfer as a determining mechanism for the local temperature in our experiments, and we identify the importance of heat outflow through the top of the catalyst bed depending on the reactor radius. The catalyst grain size and reactor dimensions are relevant experimental parameters to tune the actual catalyst temperature during the OCM process. These insights can be crucial in comparing and designing OCM technologies, thereby providing leads for the better catalytic valorization of methane resources. Our results and subsequent descriptions of the relevant heat transfer phenomena showcase the opportunities for the use of LT to quantitatively study local temperatures and effects of exothermicity and heat management *operando* in a high‐temperature catalytic reactor, in both steady‐state and transient experiments. This provides a new framework of thinking for experiment‐ and modeling‐based approaches to study challenging exothermic processes at high temperatures.

## Author Contributions

**Daniël W. Groefsema**: conceptualization, investigation, writing – original draft, methodology, validation, visualization, writing – review and editing, formal analysis, and data curation. **Freddy T. Rabouw**: supervision, writing – review and editing, formal analysis, methodology, investigation, and conceptualization. **D. Michiel Boele**: conceptualization, supervision, writing – review and editing, resources, methodology, and validation. **A.N. René Bos**: Writing – review and editing and validation. **Alexander P. van Bavel**: methodology, conceptualization, supervision, resources, writing – review and editing, and validation. **Bert M. Weckhuysen**: conceptualization, funding acquisition, writing – review and editing, supervision, resources, project administration, and methodology.

## Conflicts of Interest

The authors declare no conflicts of interest.

## Supporting information




**Supporting File**: The authors have cited additional references within the Supporting Information [49, 50, 51, 52, 53, 54, 55, 56, 57, 58, 59, 60, 61, 62, 63, 64, 65, 66, 67, 68, 69, 70, 71, 72, 73, 74, 75, 76, 77, 78, 79, 80, 81, 82, 83, 84].

## Data Availability

The data that support the findings of this study are available on request from the corresponding author. The data are not publicly available due to privacy or ethical restrictions.
