# Supplementary material for: Measuring Local Exothermic Effects During the Oxidative Coupling of Methane Using Operando Luminescence Thermometry
Source: Angew Chem Int Ed Engl. 2026 Jun 30;65(36):e1717647. doi: 10.1002/anie.1717647 (PMC13528524; doi:10.1002/anie.1717647)
Supplement: Supplementary file 1 [file ANIE-65-e1717647-s001.docx]

***Supplementary information for***

**Measuring local exothermic effects during the oxidative coupling of methane using *operando* luminescence thermometry**

*Daniël W. Groefsem**a^1^, Freddy T. Rabouw^1,2^, D. Michiel Boele^3^, Alexander P. van Bavel^3^, A.N. René Bos^3,4^, and Bert M. Weckhuysen^1,*^*

^1^ Inorganic Chemistry and Catalysis group, Institute for Sustainable and Circular Chemistry, Utrecht University, Universiteitsweg 99, 3584 CG Utrecht, The Netherlands

^2^ Soft Condensed Matter and Biophysics group, Debye Institute for Nanomaterials Science, Utrecht University, Princetonplein 1, 3584 CC Utrecht, The Netherlands

^3^ Shell Global Solutions International B.V., 1031 HW Amsterdam, the Netherlands

^4^ Laboratory for Chemical Technology, Ghent University, Technologiepark 125, 9052 Ghent, Belgium

^*^ E-mail: B.M.Weckhuysen@uu.nl

**Contents**

1.	Temperature-induced Boltzmann distribution and temperature-dependent emission intensity ratios	3

2.	Overview of earlier luminescence thermometry applications	4

3.	Determination of the spot size for measurements in the reactor	7

4.	Experimental section	9

5.	Calculation of conversion, selectivity, carbon/oxygen balance, water formation	13

6.	Integration ranges for the luminescence intensity ratios	15

7.	Heat generation due to the oxidative coupling of methane	17

8.	Schematic illustrations of heat transport processes and experimental variations affecting the local catalyst temperature	18

9.	Additional calibration data	19

10.	Calculation of thermal conductivity through the catalyst bed	20

11.	Effect of experimental parameters and local temperature on the performance for oxidative coupling of methane	23

12.	Model description	25

13.	Timescale of temperature dynamics	35

14.	Supplementary References	38

1. Temperature-induced Boltzmann distribution and temperature-dependent emission intensity ratios

The temperature-dependent emission used in this work originates from electronic transitions within the 4f orbitals of trivalent Eu ions^1,2^. After excitation, the excited state population is distributed over thermally coupled energy levels following a Boltzmann distribution:

$$\frac{N_{2}}{N_{1}}=\frac{g_{2}}{g_{1}}e^{-\Delta E/k_{\text{B}}T}$$

Equation (S1.1)

where $N_{i}$ is the population of energy level $i$, $g_{i}$ is the degeneracy of the energy level, $\Delta E$ is the energy difference between the energy levels, $k_{\text{B}}$ is the Boltzmann constant, and $T$ is the temperature. Since the emission intensity from a certain energy level is proportional to the population of that level, the intensity ratio of emission from the thermally coupled states is

$$\frac{I_{2}}{I_{1}}=Ce^{-\Delta E/k_{\text{B}}T}$$

Equation (S1.2)

where $I_{i}$ is the emission intensity from energy level $i$ and $C$ is a constant containing the degeneracies, the spontaneous emission rates, and the wavelengths of the emission. Assuming a fast distribution between the energy levels relative to the emission from those levels, which is the case for the ^5^D_0_ and ^5^D_1_ energy levels in trivalent europium ions at elevated temperatures, the intensity ratio can be used to determine the temperature of the emitting material.

2. Overview of earlier luminescence thermometry applications

Although Luminescence Thermometry (LT) materials for (potential) high-temperature applications have been reported, in some cases already decades ago^3–7^, many of these experiments use dedicated and complex optical instrumentation, which is difficult to use in an operational high-temperature reactor. This makes the application of these techniques in catalytic environments challenging. Most of the published work in the field of LT describes the synthesis, characterization, and (expected) performance of new and improved temperature-sensing materials. Actual applications of these materials and techniques are far more scarce in the published literature.

As of May 2025, only several reports have shown *operando* application of LT during catalytic reactions, up to a maximum temperature of 600 °C^8–18^ (the only exception being previous methodological work in our group^19^). Figure S1 shows an overview of reports published since 2000 where the application of LT techniques has been confirmed. In most cases, the application is limited to systems where no (catalytic) chemical conversion occurs during the experiment.

Figure S1: Overview of Luminescence Thermometry (LT) applications. White markers indicate miscellaneous LT applications, in the absence of chemical conversion processes (references in Table S1). Blue labels indicate LT applications where catalytic activity and application of LT in the same experiment was reported^8–19^. The colored background indicates the temperature ranges for low temperature applications (blue), previously reported catalytic applications (yellow), and the required temperature range for the OCM process (red)^20–25^. Last updated on March 27, 2026.

Although the first report of LT during catalytic reactions already showed a high maximum temperature of 600 °C, extending this limit for catalytic remains difficult, because of the limited thermal stability of the temperature-sensing materials and strong reduction of signal intensity at higher temperatures.

The white markers in Figure S1, labeled “miscellaneous LT applications”, refer to LT applications for processes such as microfluidic heat mixing, Joule heating, battery charging, and temperature-measurement in a reactor without chemical processes taking place (see Table S1 for the references). The blue markers in Figure S1 refer to LT applications where catalytic activity and LT in the same experiment were confirmed (references are included in the figure).

Table S1: References of the miscellaneous Luminescence Thermometry (LT) applications shown in Figure S1 (white markers).

| Reference | Date of publication | Max. temperature (°C) |
| --- | --- | --- |
| ^26^ | July 2001 | 90 |
| ^27^ | March 2006 | 87 |
| ^28^ | August 2007 | 65 |
| ^29^ | May 2008 | 400 |
| ^30^ | June 2008 | 64 |
| ^31^ | December 2010 | 62 |
| ^32^ | September 2011 | 61 |
| ^33^ | September 2013 | 227 |
| ^34^ | November 2013 | 100 |
| ^35^ | March 2016 | 40 |
| ^36^ | November 2017 | 50 |
| ^37^ | May 2018 | 500 |
| ^38^ | February 2019 | 120 |
| ^39^ | June 2019 | 400 |
| ^40^ | August 2019 | 600 |
| ^41^ | November 2020 | 105 |
| ^42^ | March 2021 | 350 |
| ^43^ | May 2023 | 227 |
| ^44^ | February 2024 | 150 |
| ^45^ | March 2024 | 70 |
| ^46^ | April 2025 | 200 |

Figure S1 does not aim to provide a complete overview of publications concerning Luminescence Thermometry. Instead, it focuses on the possibilities for application in high temperature catalytic environments. Applications far from our desired conditions have been omitted. For example, applications in physiological or liquid media were not included as "catalytic applications", although in some cases catalytic activity was reported, because of the very different nature of the application environment compared to the high-temperature gas-solid OCM process. Similarly, examples of *ex-situ* applications at high temperatures, that require complicated optical setups, have not been included due to the limited applicability of such a setup for *operando* measurements in a reactor. The overview was constructed using the Google Scholar search engine, by searching for the words “*operando* luminescence thermometry” (last updated on March 27, 2026).

3. Determination of the spot size for measurements in the reactor

The precise resolution of the measurements presented in the main text is difficult to assess, due to the complicated optical paths through multiple curved interfaces of the reactor tube and the complex scattering/absorption processes in a packed catalyst bed.

The probe used in this work produces a divergent beam. We manually estimate the measured axial spot size by changing the optical probe height from above the catalyst bed downward, while continuously monitoring the collected emission. Aligned above the catalyst bed, no europium emission is observed in the monitored emission spectra. We note the height at which the measurement spot starts to reach the catalyst bed, indicated by the observation of europium emission in the monitored spectra (point 1). We then continue to move the probe downward, which increases the intensity of recorded europium emission, because an increasing part of the spot is aligned with the emitting catalyst bed. We note the height at which the collected europium emission intensity does not increase with further downward movement of the probe anymore (point 2). Point 2 indicates the probe height at which the spot is fully aligned with the emitting material in the catalyst bed. The height difference between point 1 (onset) and point 2 (full alignment) is the manual estimation of the axial spot size; determined to be ~ 1.5 mm.

To confirm the estimation of the ~ 1.5 mm axial spot size, determined manually, we calculate the theoretical spot size based on the alignment and optical properties of the probe and reactor tube material. Figure S2 shows a schematic overview of the relevant refraction interfaces and their effect on the axial spot size. Using *NA* = 0.22 for the probe tip, as reported by the supplier, Snell’s law of refraction, an estimated probe-reactor distance of 1 mm, and a measured reactor wall thickness of 1.4 mm, we calculate a theoretical axial spot size of ~ 1.3 mm, which is in acceptable agreement with the manually determined axial spot size of 1.5 mm. We take the manually determined 1.5 mm as upper limit of the axial spot size mentioned in the main text.

Figure S2: Schematic overview of the estimation of the theoretical axial spot size for the measurements presented in the main text, using the diameter of the read fiber d = 0.4 mm as reported by the supplier, the numerical aperture of the probe NA = 0.22 as reported by the supplier, the refractive index of air n_1_ = 1.000274 at standard temperature and pressure^47^, and the refractive index of the quartz reactor tube n_2_ = 1.4585 as reported by the supplier.

4. Experimental section

All the catalytic tests and *operando* thermometry experiments were performed in an electrically heated fixed-bed quartz tube plug flow reactor equipped with in-line gas chromatography. Details of the setup can be found in Section 4.1. Details of the experimental methods can be found from Section 4.2 onwards.

4.1. Details of the reactor setup

Figure S3: Pictures and schematic overview of the reactor setup.

A quartz reactor tube is placed vertically in an in-house designed oven with a slit on one side for optical access to the reactor tube. A K-type thermocouple is fixated to the reactor tube using metal wire, with the tip placement corresponding to the axial alignment of the optical probe. Reactant flows of CH_4_, O_2_, N_2_, and He (Linde 5.0) are controlled with digital mass flow controllers (MFC’s: Bronkhorst EL-FLOW Prestige FG-201CV). Reaction products are analyzed with a gas chromatograph (Global Analyser Solutions CompactGC4.0) equipped with an FID channel for hydrocarbon (C_1_–C_5_) products and a TCD channel for permanent gases (He, H_2_, N_2_, O_2_, CO, CO_2_, and CH_4_). The product mixtures are separated prior to detection using Restek Rt-TCEP + Rtx-1 and Al2O3/Na2SO4 columns for the hydrocarbon channel and Rt-Q-Bond and Carbonxen1010 columns for the permanent gases. Heat traced gas lines are heated to 130 °C to prevent condensation of water.

4.2. Catalytic testing and oven temperature control

In a typical experiment, 0.50 g of 4 at% Eu-doped Y_2_O_3_ (99%, Sigma-Aldrich) was pressed and sieved to the desired size. The catalyst material was loaded in a quartz reactor tube and calcined *in-situ* at 700 °C under a 20% O_2_ in N_2_ flow for 3 h prior to experiments. The oven temperature was controlled and/or monitored with a thermocouple fixated to the outside of the quartz reactor tube. For the catalytic tests, the oven was heated to the desired temperature and the catalytic activity was measured after a stable temperature and conversion were reached. For the thermometry experiments, the oven temperature was controlled via a constant heating power instead of a temperature set point, to prevent feedback from the exothermic reactions. The oven temperature was stabilized overnight at constant heating power to ensure a constant oven temperature prior to experiments. Steady-state temperature measurements were performed after a stable temperature and conversion was reached. For experiments where reactant feeds were varied, a back check was performed to eliminate possible changes in the alignment or catalyst material during the experiments.

4.3. Europium emission measurements

Spectroscopic measurements were performed using a square-wave-modulated laser from CNI Optoelectronics Technology (λ = 392 nm, MDL-NS-395nm-100mW, 2 kHz pulse frequency) coupled into an Avantes High Temperature Reflection Probe (FCR-7UVIR400-2.5-BX-6X350-HTX) for excitation and signal collection. The signal was measured with an Avantes AvaSpec spectrometer (AvaRaman-532 TEC) for *in-situ*/*operando* measurements or an Avantes Starline spectrometer (AvaSpec-ULS2048CL-EVO-RS) for *ex-situ* measurements, using AvaSoft 8 software. A 400 nm in-line long-pass filter was placed in the detection path to protect the detector from laser exposure. *In-situ* and *operando* measurements were performed by aligning the optical probe to the quartz reactor tube through a small slit in the oven. The optical probe was manually aligned to the top of the catalyst bed, unless stated otherwise. The *ex-situ* measurements were performed in a Linkam TS1000 heating stage.

4.4. Calibration and processing of temperature-dependent europium emission

Temperature-dependent europium emission was calibrated by measuring emission spectra of the catalyst material at controlled temperatures in the reactor under inert atmosphere without gas flow. An *in-situ* calibration was measured for each experiment in the reactor, to correct for differences in alignment and scattering, affecting the temperature-independent calibration constant. For each calibration and temperature measurement, 50 emission spectra were recorded, and 50 reference spectra (“dark”, without laser excitation) were averaged for background correction of the corresponding emission spectra. We expect that systematic errors are significant for the *operando* temperature measurements and that analyses with different integration ranges will be affected differently by the systematic error. The luminescence intensity ratio (LIR) of emission from the ^5^D_1_ and ^5^D_0_ energy levels is obtained from each corrected spectrum using five different integration ranges, and subsequently converted into a read-out temperature via a fit of the corresponding calibration curve to the LIR, yielding five sets of 50 values of *T*_read-out_ per reported temperature. For each integration range, the 50 read-out temperatures are averaged, yielding five average values of *T*_read-out_ per measurement. The final reported temperature is the average of these five values, taking the smallest and largest values as an estimate for the systematic uncertainty on the measurements. The final random temperature error in the measurement $\sigma_{T,\text{random}}$ is estimated as the root-mean-square (RMS) deviation for each recorded temperature over the 50 spectra using the different integration ranges:

$$\begin{aligned} \sigma_{T,\text{random}}=\sqrt{\frac{1}{5}\left( \sigma_{\text{A}}^{2}+\sigma_{\text{B}}^{2}+\sigma_{\text{C}}^{2}+\sigma_{\text{D}}^{2}+\sigma_{\text{E}}^{2} \right)} \end{aligned}$$

Equation (S4.1)

with $\sigma_{i}^{2}$ the standard deviation in the 50 read-out temperatures for the five different integration ranges $i$ = 1–5 (see also Section 6, SI).

The catalyst temperature varies strongly in the different experiments and is largely unknown beforehand. Because the emission intensity is affected by the temperature of the emitting material, this can lead to a very low emission intensity, or to saturation of the detector, when the excitation power is too low or too high. To deal with the large variations in emission intensity, the measurements are performed with two different excitation powers (i.e., different duty cycles of the excitation pulse). This makes it possible to select measurements with a high recorded emission intensity and circumvent detector saturation in most cases. Calibration measurements are performed after the catalytic experiments, with a calcination step prior to the calibration. The excitation settings for the calibrations are the same as selected in the catalytic experiments to allow for accurate calibration. In some cases, even the lowest excitation power turned out to lead to saturation of the detector, due to the very high catalyst temperatures during the catalytic experiments. In these cases, the spectra in the integration range that contains the saturated emission peak were omitted from the analyses.

The time-resolved temperature measurements in Figure 8 in the main text were performed with a single emission spectrum for each measurement, using the average of 50 dark spectra at the end of the measurement for the background corrections. This can cause overcorrection and thereby underestimation of the initial temperatures in these measurements.

Additional calibration data for the experiments described in the main text are shown in Section 9, SI.

5. Calculation of conversion, selectivity, carbon/oxygen balance, water formation

The gas chromatograph (GC) is calibrated using a mixture containing C_1_–C_5_ n-paraffins and olefins and all detected permanent gases.

5.1. Conversion

CH_4_ and O_2_ conversions are calculated according to

$$X_{i}=\left( 1-\frac{x_{i,\mathrm{out}} ISC}{x_{i,in}} \right)\times100\%$$

Equation (S5.1)

with $X_{i}$ the conversion of component $i$, $x_{i, in}$ and $x_{i, out}$ the volumetric concentration of component $i$ at the inlet and outlet, respectively, and $ISC$ the internal standard correction calculated as the ratio $ISC=x_{standard, in}/x_{standard, out}$ of the internal standard concentration in the reactant and product mixtures.

5.2. Selectivity

Molar selectivities $S_{j}$ towards products $j$ are based on CH_4_ conversion and calculated according to

$$S_{j}=\frac{n_{j}x_{j,out} ISC}{x_{CH_{4},in}-(x_{CH_{4},out} ISC)}\times100\%$$

Equation (S5.2)

with $n_{j}$ the number of carbon atoms in product $j$.

5.3. Carbon and oxygen balance

Carbon and oxygen balances are calculated according to

$$carbon balance=\frac{\sum_{i} n_{i}x_{i,out} ISC}{x_{CH_{4},in}}\times100\%$$

Equation (S5.3)

and

$$oxygen balance=\frac{\sum_{j} n_{j}x_{j,out} ISC}{{2x}_{O_{2},in}}\times100\%$$

Equation (S5.4)

where the summations run over all carbon-containing components $i$ and oxygen-containing components $j$ in the product mixture, containing $n_{i}$ atoms of carbon or $n_{j}$ atoms of oxygen, respectively. The carbon balance lies within 95–105% for all experiments. The oxygen balance is used to determine the amount of water that is formed in the process, as explained below.

5.4. Water formation

The GC used is not able to detect water. Since O_2_ and other oxygen-containing compounds are detected, the volumetric concentration of water in the product $x_{water, out}$ can be calculated from the oxygen that is unaccounted for, via

$$x_{water,out}=2x_{O_{2},in}{(100\%-OB)}/{100\%}$$

Equation (S5.5)

with $x_{O_{2},in}$ the volumetric concentration of O_2_ in the reactant mixture, and $OB$ the oxygen balance (%), corrected for two oxygen atoms in O_2_. As a control, the hydrogen balance is calculated for the full product mixture including the calculated H_2_O, similar to the carbon and oxygen balances in Equations (S5.3) and (S5.4). The hydrogen balance lies within 95–105% for all experiments.

6. Integration ranges for the luminescence intensity ratios

Each calibration and temperature measurement consists of recording 50 emission spectra with two different excitation settings (i.e., different duty cycles of the excitation pulse) and 50 reference spectra (“dark”, without laser excitation) to correct for thermal background radiation. All corrected spectra were processed with varying integration ranges to obtain the luminescence intensity ratios (LIR). To estimate the magnitude of the systematic error, we calculate the LIR and corresponding $T_{\text{read-out}}$ for each of the 50 spectra per temperature measurement, with varying integration ranges A–E, as shown in Figure S4 and summarized in Table S2. All panels in Figure S4 show a background-corrected emission spectrum recorded at $T_{\text{oven}}$ = 650 °C under static N_2_ for reference. The highest intensity peak, centered around $\lambda$ = 612 nm, is outside of the plotted range for clarity.

Figure S4: Different integration ranges for the luminescence intensity ratios of europium emission from the ^5^D_1_ and ^5^D_0_ energy levels. Panels **a–e** (top to bottom) show the integration ranges A–E, as summarized in Table S2 and indicated with the same colors per integration range as Fig. 3 in the main text.

Table S2: Integration ranges for the luminescence intensity ratios of europium emission from the ^5^D_1_ and ^5^D_0_ energy levels.

| **Integration range** | **^5^D_1_ range (nm)** | **^5^D_0_ range (nm)** |
| --- | --- | --- |
| **A** (red) | 536–572 | 604–640 |
| **B** (orange) | 536–572 | 626–656 |
| **C** (yellow) | 536–572 | 626–640 |
| **D** (green) | 536–558 | 626–640 |
| **E** (blue) | 545–572 | 626–640 |

For each reported temperature, measurements were performed with two different excitation settings. For each of these settings, and for each of the 5 integration ranges shown in Figure S4, a calibration curve is fitted to the calibration measurements separately (as shown in Fig. 3 of the main text and the additional calibration data in Figure S6 in Section 9, SI). Integration range A includes the highest intensity peak centered around $\lambda$ = 612 nm, which is the one that can saturate the detector first. If this is the case in the calibration measurements, integration ranges B–E are used for the calibration and temperature measurement.

The reported temperatures in the main text are the averages of the five analyses with different integration ranges, as explained above. We take the smallest and largest temperature values obtained this way as an estimate of the systematic uncertainty. The random errors are estimated by taking the root-mean-square deviation for each set of 50 recorded temperatures from the analyses with five integration ranges, as explained in the Methods of the main text.

7. Heat generation due to the oxidative coupling of methane

The total heat generation $Q$ (unit W) of the oxidative coupling of methane (OCM) process is calculated from the known reactant input and the product output measured with gas chromatography (GC) according to

$$Q=-\Delta H_{\mathrm{rxn}}^{0}=\sum_{i}^{\mathrm{reactants}} \Delta H_{f,i}^{0} f_{i}-\sum_{j}^{\mathrm{products}} \Delta H_{f,j}^{0} f_{j}$$

Equation (S7.1)

with $\Delta H_{f,i}^{0}$ and $\Delta H_{f,j}^{0}$ the standard enthalpies of formation (in J mol^−1^) of components $i$ and $j$ in the reactant and product mixture, respectively; and $f_{i}$ and $f_{j}$ the flows (in mol s^−1^) of reactants $i$ and products $j$, respectively. The values for $\Delta H_{f}^{0}$ are taken from the NIST Chemistry WebBook^48^ and are shown in Table S3.

Table S3: Standard enthalpies of formation for the observed OCM reactants and products^48^.

| **Compound** | $\boldsymbol{\Delta}\boldsymbol{H}_{\mathbf{f}}^{\boldsymbol{0}}$ (J mol^−1^) |
| --- | --- |
| **CH_4_ (methane)** | −74 600 |
| **C_2_H_6_ (ethane)** | −84 000 |
| **C_2_H_4_ (ethylene)** | +52 400 |
| **C_3_H_8_ (n-propane)** | −104 700 |
| **C_3_H_6_ (c-propane)** | +53 300 |
| **C_3_H_6_ (propylene)** | +20 410 |
| **CO (carbon monoxide)** | −110 530 |
| **CO_2_ (carbon dioxide)** | −393 520 |
| **H_2_O (water)** | −241 830 |
| **O_2_ (oxygen)** | 0 (reference state) |
| **H_2_ (hydrogen)** | 0 (reference state) |

8. Schematic illustrations of heat transport processes and experimental variations affecting the local catalyst temperature

Figure S5: **a**, Schematic illustration of the heat transport processes affecting the local catalyst temperature. **b**, Schematic illustrations of the varied experimental parameters.

9. Additional calibration data

Figure S6: Calibration data for the experiments presented in the main text, with 2R = 4.1, d_p_ = 300 μm, 10% He (left column), 2R = 4.1, d_p_ = 732 μm, 10% He (middle column), and 2R = 4.1, d_p_ = 300 μm, 50% He (right column). Rows are separated for measurements in the top and middle of the catalyst bed using different excitation duty cycles, labeled on the right of each row with “top” and “mid” for the measurement position, and “low” and “high” for the excitation duty cycle. Integration range A (red, see Table S2 in Section 6, SI) is omitted for measurements where the calibration spectra were saturated in the corresponding wavelength range.

10. Calculation of thermal conductivity through the catalyst bed

The calculations for thermal conductivity through the catalyst bed are based on Yagi and Kunii^49^, Kunii and Smith^50^, and Zehner and Schlünder^51^, according to expressions by Froment, Bischoff, and De Wilde^52^ for the static thermal conductivity $\lambda_{\mathrm{static}}$.

10.1. Calculations of static conductivity

The following expressions are used to calculate the static thermal conductivity based on Yagi/Kunii and Kunii/Smith:

$$\lambda_{static,Kunii}=\lambda_{g}\left( \varepsilon+\frac{d_{p}\alpha_{\mathrm{rv}}}{\lambda_{g}}+\frac{0.9(1-\varepsilon)}{\phi+\frac{2}{3}\frac{\lambda_{g}}{\lambda_{s}}} \right)$$

Equation (S10.1)

with

$$\alpha_{\mathrm{rv}}=\frac{0.227}{1+\frac{\varepsilon}{2(1-\varepsilon)}\frac{1-p}{p}}\left( \frac{T}{100} \right)^{3}$$

$$\phi=\phi_{2}+({\phi_{1}-\phi}_{2})\frac{\varepsilon-0.260}{0.476-0.260}$$

Equations (S10.2)

The following expressions are used to calculate the static thermal conductivity based on Zehner/Schlünder:

$$\lambda_{static,Zehner}=\lambda_{g}\left( \left( 1-\sqrt{1-\varepsilon} \right)\left( 1+\varepsilon\Omega\right)+\frac{2\sqrt{1-\varepsilon}}{1+\left( \Omega-B \right)\frac{\lambda_{g}}{\lambda_{s}}}\Theta\right)$$

Equation (S10.3)

with

$$\Omega=\frac{{d_{p}\alpha}_{\mathrm{rs}}}{\lambda_{g}}$$

$$\alpha_{\mathrm{rs}}=0.227\frac{p}{2-p}\left( \frac{T}{100} \right)^{3}$$

$$\Theta=\frac{\left( 1+\left( \Omega-1 \right)\frac{\lambda_{g}}{\lambda_{s}} \right)B}{\left( 1+\left( \Omega-B \right)\frac{\lambda_{g}}{\lambda_{s}} \right)^{2}}\ln\left( \frac{1+\Omega}{B\frac{\lambda_{g}}{\lambda_{s}}} \right) -\frac{B-1}{1+\left( \Omega-B \right)\frac{\lambda_{g}}{\lambda_{s}}}+\frac{B+1}{2B}\left( \Omega-B \right)$$

$$B=b\left( \frac{1-\varepsilon}{\varepsilon} \right)^{10/9}$$

Equations (S10.4)

Meanings of the symbols are given in Table S4 and explanations of our assumptions are given below.

Table S4: Parameters in the calculations for the static thermal conductivity through the catalyst bed.

| **Parameter** | **Explanation** |
| --- | --- |
| $T$ | Temperature (K). The temperature varies within experiments. *T*-dependent parameters are calculated at the representative temperature of 670 °C. |
| $\lambda_{g,s}$ | Thermal conductivity of gas or solid (W m^−1^ K^−1^) |
| $\varepsilon$ | Void fraction (${V_{\mathrm{voids}}}/{V_{\mathrm{catalyst}}}$) |
| $d_{p}$ | Catalyst particle diameter (m) |
| $\alpha_{\mathrm{rv}}$ | Radiation coefficient from void to void (J m^−2^ s^−1^ K^−1^) |
| $\alpha_{\mathrm{rs}}$ | Radiation coefficient for the solid (J m^−2^ s^−1^ K^−1^) |
| $\phi$ | Depends on the packing density, calculated from $\phi_{1}$ and $\phi_{2}$ |
| ${\phi_{1},\phi}_{2}$ | Value of $\phi$ for the loosest and densest packing ($\varepsilon$= 0.476, 0.260), taken from ${\lambda_{s}}/{\lambda_{g}}$ according to Froment, Bischoff, and De Wilde^52^ |
| $p$ | Emissivity of the solid (between 0–1) |
| $b$ | 1.25 for spheres^52^ |

10.2. Contribution of convective heat transfer

When a gas flow is applied, thermal conduction via the gas flow can increase the effective thermal conductivity $\lambda_{\mathrm{eff}}$ by adding a convective contribution to $\lambda_{\text{static}}$. $\lambda_{\mathrm{eff}}$ will be proportional to the gas phase density, velocity, heat capacity, and particle diameter, and can be approximated as:

$$\lambda_{\mathrm{eff}}=\lambda_{\mathrm{static}}+0.1\rho_{g}v_{g}C_{p,g}d_{p}$$

Equation (S10.5)

Meanings of the symbols are given in Table S5 and explanations of our assumptions are given below. The factor 0.1 in Equation (S10.5) is a convective correlation constant, assumed to be on the order of 0.1. Varying this constant over two orders of magnitude yields consistent results: the calculated $\lambda_{\text{eff}}$ varies within only 2%, showing the small contribution of convection in our conditions consistent with our experimental observations. We do not attempt to further estimate this coefficient.

Table S5: Parameters for the convective contribution to the effective thermal conductivity.

| **Parameter** | **Explanation** |
| --- | --- |
| $\rho_{g}$ | Density of the gas (mol m^−3^) |
| $v_{g}$ | Gas velocity (m s^−1^) |
| $C_{p,g}$ | Heat capacity of the gas (J mol^−1^ K^−1^) |
| $d_{p}$ | Catalyst particle diameter (m) |

10.3. Assumptions and uncertainty in calculated values

Accurate calculation of the effective thermal conductivity through the catalyst bed is not trivial and requires detailed knowledge of all parameters. Our assumptions are shown in Table S6. These assumptions allow us to extract relative changes in thermal conductivity by varying the relevant parameters. Agreement between the two models for static thermal conductivity is relatively poor in our calculations (within 23%), but the convective contribution to the effective thermal conductivity is always < 2.5%, and agreement between the two models for relative changes of the effective thermal conductivity with changing experimental parameters is within 5%.

The outcome of the calculations depends on the input value $\lambda_{\mathrm{solid}}$ for Y_2_O_3_, for which a large variation exists in reported values^53–56^. Taking the smallest and largest reported values for $\lambda_{\mathrm{solid}}$ and using both models shown above, for all experimental conditions, we calculate a range of values $\lambda_{\mathrm{eff}}$ = 0.2–2.6 W m^−1^ K^−1^.

Table S6: Assumptions for the calculations of static and effective thermal conductivity.

| **Parameter** | **Assumption** |
| --- | --- |
| *T* | The temperature varies within experiments. Temperature-dependent parameters are calculated at the representative temperature of 670 °C. |
| $\rho_{g}$ | Density of the gas is calculated as a weighted average of the individual densities of the reactant gas mixture. |
| $v_{g}$ | Gas velocity is calculated for unperturbed (laminar) flow as the volumetric flow rate divided by the cross-sectional area of the reactor tube. |
| $C_{p,g}$ | Heat capacity of the gas is calculated as a weighted average of the individual heat capacities of the reactant gas mixture. |

11. Effect of experimental parameters and local temperature on the performance for oxidative coupling of methane

Due to the mutual interaction between temperature, reaction kinetics, and heat generation, it is difficult to study the influence of a single parameter on the OCM performance. The local temperature, conversion, selectivity, and heat generation vary between the experiments, and the measured temperature is often not representative for the whole catalyst bed, making e.g. comparison at constant methane conversion or at constant temperature difficult. Despite this, several trends are indicated concerning the OCM performance in our experiments. Figure S7 shows the methane and oxygen conversion and selectivity towards C_2_ and CO_x_ products as a function of the catalyst temperature in the top of the catalyst bed.

Figure S7: OCM performance as a function of catalyst temperature, for the variations in reactor diameter, catalyst grain size, and inert diluent concentration from the main text. **a**, Methane conversion, **b**, selectivity towards C_2+_ products, **c**, selectivity towards CO_x_ products, **d**, oxygen conversion, **e**, the ratio of ethylene/C_2+_ in the product mixture, **f**, the ratio of CO/CO_x_ in the product mixture.

Although some variations are observed, generally, there is a correlation of higher methane conversion, C_2+_ selectivity, and ethylene/C_2+_ ratio with higher catalyst temperature under the studied reaction conditions. Furthermore, the selectivity towards C_2+_ products is higher for the reactor tube with the larger inner diameter of 7.1 mm (compared to the narrower reactor tube at similar catalyst temperatures), accompanied by a slightly larger ethylene/C_2_ ratio, under most conditions. This may be attributed to the detrimental effect of hot spot formation, which is more pronounced in the narrow reactor tube, causing larger contributions of unselective high temperature gas phase reactions. The trend of increasing ethylene/C_2_ with catalyst temperature is consistent with the expected increase in ethane dehydrogenation at higher temperatures.

12. Model description

We use a cylindrical coordinate system for calculations on the reactor. The reactor has a radius $R$. The region between $0<z<L$ contains the catalyst bed, while the region $z<0$ contains quartz wool. The exothermic OCM process generates heat in the catalyst bed at a rate per unit of volume $W(\rho,z)$ (unit W m^−3^). This creates a temperature profile $T(\rho,z)$. Heat diffuses through the catalyst bed from regions of higher to regions of lower temperature and eventually escapes from the reactor wall to the oven. We assume that the catalyst bed and the layer of quartz wool have the same temperature- and position-independent heat conductivity $k$ (unit W m^−1^ K^−1^). The heat flux (unit W m^−2^) is

$$\mathbf{q}=-k\nabla T$$

Equation (S12.1)

Upon constant reactant influx, the system will eventually reach a steady-state situation with a stable temperature profile. The steady-state temperature profile must obey

$$\nabla\cdot\mathbf{q}=W$$

Equation (S12.2)

Heat is not generated homogeneously throughout the catalyst bed. The oxygen conversion is over ~ 50% in all experiments presented in the main text and over 95% in several. This indicates that the reaction rate, and hence the heat generation, must be lower at the bottom of the catalyst bed than at the top. For this model, we neglect positive feedback of heat generation on the reaction rate, which in reality will affect the final heat generation profile. We assume that the heat generation rate is independent of $\rho$, neglecting any radial flow profile, and that the heat generation decays exponentially with $z$:

$$W\left( z \right)=W_{0}e^{-z/d}$$

Equation (S12.3)

We obtain an estimate for the length scale $d$, over which the heat generation rate decreases with $z$, from the oxygen conversion $X_{O_{2}}$ as measured with GC. The estimate assumes that the reaction rate at the bottom of the catalyst bed ($z=L$) is lower than at the top by a factor $1/\left( 1-X_{O_{2}} \right)$, essentially assuming that the reaction is first-order in O_2_ and zero-order in everything else:

$$d=\frac{L}{\ln\left( \frac{1}{1-X_{O_{2}}} \right)}$$

Equation (S12.4)

From the full composition of the input and output streams of the reactor, based on GC analysis, we determine the heat of reaction $Q$ (unit W) integrated over the catalyst bed (Section 7 of the Supplementary Information). This number is related to the heat generation rate per unit of volume as

$$Q=\pi R^{2}\int_{0}^{L} W\left( z \right) dz$$

Equation (S12.5)

Solving for $W_{0}$ yields

$$W_{0}=\frac{Q}{\left( d-d e^{-L/d} \right)\pi R^{2}}=\frac{Q\ln\left( \frac{1}{1-X_{O_{2}}} \right)}{X_{O_{2}}\pi R^{2}L}$$

Equation (S12.6)

Equations (S12.3), (S12.4), (S12.6) provide a simple model for the position-dependent heat generation rate as a function of the catalyst bed dimensions $R$ and $L$, and as a function of the reaction results as quantified by $Q$ and $X_{O_{2}}$.

Our models presented below aim at explaining the temperature $T_{\mathrm{rec}}$ recorded in a luminescence-thermometry experiment in terms of the reactor design and the reaction results $Q$ and $X_{O_{2}}$. While $Q$ and $X_{O_{2}}$ in turn depend on the composition and flow rate of the input gas mixture, we do not attempt to calculate or predict this dependence, instead taking $Q$ and $X_{O_{2}}$ as input parameters for our models.

12.1. Model 1: neglect heat diffusion in the $\boldsymbol{z}$ direction

In model 1, we calculate steady-state temperature gradients in the $\rho$ direction using Equation (S12.2). If we neglect heat diffusion in the $z$ direction, i.e., we set $q_{z}=0$, Equations (S12.1) and (S12.2) in cylindrical coordinates yield

$$-k\left( \frac{\partial^{2}T}{\partial\rho^{2}}+\frac{1}{\rho}\frac{\partial T}{\partial\rho} \right)=W$$

Equation (S12.7)

where $W$ is the heat generation rate per unit of volume at the height $z$ under consideration. The general solution to Equation (S12.7) is

$$T\left( \rho\right)=-\frac{W}{4k}\rho^{2}+C_{1}\ln\left( \rho\right)+C_{2}$$

Equation (S12.8)

where $C_{1}$ and $C_{2}$ are integration constants. We apply the boundary conditions that (i) $T(0)$ must be finite, and (ii) the radial heat flux at the reactor wall ($\rho=R$) matches the heat flux towards the environment. Boundary condition (i) requires that

$$C_{1}=0$$

Equation (S12.9)

We express boundary condition (ii) as

$$-k\left. \frac{\partial T}{\partial\rho} \right|_{\rho=R}=\alpha[T\left( R \right)-T_{\mathrm{oven}}]$$

Equation (S12.10)

where $T_{\mathrm{oven}}$ is the external oven temperature and $\alpha$ (unit W m^−2^ K^−1^) is the coefficient for heat transfer through the reactor wall and into the oven. Solving equation (S12.10) for $C_{2}$ yields

$$C_{2}=T_{\mathrm{oven}}+\frac{W}{4k}R^{2}+\frac{W}{2\alpha}R$$

Equation (S12.11)

The precise spatial resolution of the experimental measurements is difficult to assess. To facilitate the modeling, we assume that the luminescence thermometry experiment records the temperature $T_{\mathrm{rec}}$ averaged over the area of a slice through the catalyst bed at constant $z$:

$$T_{\mathrm{rec}}=\left( \pi R^{2} \right)^{-1}\int_{0}^{R} T\left( \rho\right)2\pi\rho d\rho=T_{\mathrm{oven}}+\frac{W}{4k}R^{2}+\frac{W}{2\alpha}R$$

Equation (S12.12)

We expect that, in reality, the measurement will record an average temperature over the axial spot at a certain height $z$, weighted by the varying total emission intensity with local temperature (i.e., higher intensity signal for regions with lower temperature within the measured spot, leading to an underestimation of the actual catalyst temperature at height $z$). This will result in a smoothing of the axial temperature profiles that are shown below, but not a fundamentally different profile.

Equation (S12.12) describes the locally recorded temperature $T_{\mathrm{rec}}(z)$ at height $z$ in the catalyst bed, depending on the local heat generation rate $W(z)$ at the same location as given by Equations (S12.3), (S12.4), (S12.6) and in the absence of heat diffusion in the $z$ direction.

In Figure S8, we fit the experimental values of $T_{\mathrm{rec}}$ to Equation (S12.12). Our global fit procedure uses all 27 temperature recordings for $d_{p}$ = 300 µm simultaneously, taking the catalyst bed dimensions $R$ and $L$ (systematically controlled) and reaction results $Q$ and $X_{O_{2}}$ (experimentally determined) as input parameters and fitting for the best values of $k$ and $\alpha$ as well as the location $z_{0}$ of the measurements. The model matches the experimental temperatures to within a root-mean-square deviation of $\sqrt{\left\langle\delta T^{2} \right\rangle}$ = 8 °C. It reproduces the qualitative trends of higher temperatures in the narrow reactor (Figure S8b) than in the wide reactor (Figure S8a) and lower temperatures in the middle of the catalyst bed (Figure S8c) compared to the top (Figure S8b).

Figure S8: Plots of the local excess catalyst temperature during the OCM process as a function of the heat generation (x axis) and oxygen conversion (y axis). The disks show the recorded excess catalyst temperature in the experiments, relative to the constant oven temperature under inert conditions. The background maps show model 1 (Equation (S12.12)), with fitted values of $k$ = $\infty$, $\alpha$ = 2.5 × 10^2^ W m^−2^ K^−1^, and $z_{0}$ = 2.8 mm, for experiments with **a**, 2$R$ = 7.1 mm measured in the top of the catalyst bed, **b**, 2$R$ = 4.1 mm measured in the top of the catalyst bed, **c**, 2$R$ = 4.1 mm measured 9.5 mm lower in the catalyst bed. All panels use a common color scale.

Figure S9: **a**, Profile of the heat generation rate $W(z)$ per unit of volume for selected experiments i and ii in Figure S8. $W(z)$ was the input for the fit model. The decay along $z$ is assumed exponential and determined by experimental values of $Q$ and $X_{O_{2}}$. The heat generation profile along $x$ (a cross section of the catalyst bed), is uniform by assumption. **b**, Profile of the temperature $T(\rho,z)$ according to the fit to model 1. The $z$ dependence is the same as for $W(z)$ but scaled with a pre-factor that depends on $R$, $k$, and $\alpha$. The uniformity along $x$ is not imposed by the model but a consequence of the large best-fit value for $k$.

Figure S9 shows the corresponding heat generation and temperature profiles as they follow from the fits of experimental data to model 1, specifically for experiments i and ii highlighted in Figure S8. The best-fit value of $k$ yields a flat radial temperature profile (Figure S9b). This means that the initial approximation of neglecting heat diffusion in the $z$ direction was not justified.

12.2. Model 2: infinitely fast heat diffusion in the $\boldsymbol{\rho}$ direction

For model 2, we assume that the heat diffusion is sufficiently fast, such that the steady-state radial profile is flat. This assumption is based on the results of model 1 (Figure S8, Figure S9). Heat transfer through the reactor wall and into the oven (with coefficient $\alpha$) is then the rate-determining step in heat dissipation. We define the heat loss rate (unit W m^−3^ K^−1^) by transfer through the reactor wall at height $z$ as

$$\gamma=C\frac{dQ\left( z \right)}{dU\left( z \right)}=C\frac{2\pi R\alpha\left[ T\left( z \right)-T_{\mathrm{oven}} \right]dz}{\pi R^{2}C\left[ T\left( z \right)-T_{\mathrm{oven}} \right]dz}=\frac{2\alpha}{R}$$

Equation (S12.13)

where $dU(z)$ is the energy content (unit J) of an infinitely thin slice of the catalyst bed between heights $z$ and $z+dz$, $dQ(z)$ is the heat outflow (unit W) through the reactor wall of the same slice, and $C$ is the volumetric heat capacity (J K^−1^ m^−3^) of the materials inside the reactor. To simplify the modeling work, we assume that $C$ an $k$ are the same for the catalyst and the quartz wool. Although this is not fully realistic, it is questionable whether treating them separately will improve the work: it is difficult to obtain a reasonable estimate of the quartz wool thermal conductivity, because it will be significantly different than for just solid quartz, and strongly dependent on for example how firmly it is pressed when manually loading the reactor tube.

The temperature profile along the $z$ direction is described by

$$-k\frac{\partial^{2}T}{\partial z^{2}}=-\gamma(T-T_{\mathrm{oven}})+W$$

Equation (S12.14)

Inside the catalyst bed we use the exponential model $W=W_{0}e^{-z/d}$ as described above in Equations (S12.3), (S12.4), (S12.6), leading to the solution

$$T_{\mathrm{cat}}\left( z \right)=T_{\mathrm{oven}}+\frac{d^{2}e^{-z/d}}{k-\gamma d^{2}}W_{0}+C_{3}e^{\sqrt{\gamma/k} z}+C_{4}e^{-\sqrt{\gamma/k} z}$$

Equation (S12.15a)

In the quartz wool, $W$ = 0, so we obtain

$$T_{\mathrm{qw}}\left( z \right)=T_{\mathrm{oven}}+C_{5}e^{\sqrt{\gamma/k} z}+C_{6}e^{-\sqrt{\gamma/k} z}$$

Equation (S12.15b)

Four boundary conditions determine the values of integration constants $C_{3}$, $C_{4}$, $C_{5}$, and $C_{6}$. Setting (i) $\lim_{z\to-\infty} T_{\mathrm{qw}}\left( z \right)=T_{\mathrm{oven}}$ yields

$$C_{6}=0$$

Equation (S12.16)

Using this boundary condition, we treat the quartz wool layer as infinitely extended into the negative $z$ direction, while in reality it is just a few mm high. The boundary conditions (ii) $T_{\mathrm{qw}}(0)=T_{\mathrm{cat}}(0)$, (iii) $q_{z,qw}\left( 0 \right)=q_{z,cat}(0)$, and (iv) $q_{z,cat}\left( L \right)=\alpha\left[ T_{\mathrm{cat}}\left( L \right)-T_{\mathrm{oven}} \right]$ yield three equations for the three unknowns $C_{3}$, $C_{4}$, and $C_{5}$. These can be solved:

$$\begin{matrix} C_{3}= & \frac{d[2Ae^{-L\left( A+d^{-1} \right)}\left( k-\alpha d \right)-\left( 1+Ad \right)e^{-2AL}(kA-\alpha)]}{2kA(A^{2}d^{2}-1)(kA+\alpha)}W_{0} \\ C_{4}= & \frac{d}{2kA(1-Ad)}W_{0} \\ C_{5}= & \frac{d^{2}}{k(A^{2}d^{2}-1)}W_{0}+C_{3}+C_{4} \end{matrix}$$

Equation (S12.17)

with $A=\sqrt{\gamma/k}=\sqrt{2\alpha/kR}$ (unit m^−1^). Boundary conditions (ii) and (iii) describe continuity of temperature and heat flux across the interface between catalyst bed and quartz wool, while (iv) describes that the heat flux at the catalyst’s bottom surface flows out through the quartz frit into the environment with the same heat transfer coefficient $\alpha$ as for the side walls.

We perform a global fit of model 2 as given by Equations (S12.15), (S12.16), (S12.17) to all 27 experimental measurements with $d_{p}$ = 300 µm. If the location $z_{0}$ of the measurements is treated as a free parameter, the fit converges to a more unrealistic value of $z_{0}$ = 4.1 mm with $\sqrt{\left\langle\delta T^{2} \right\rangle}$ = 5 °C. We therefore fix $z_{0}$ at a more realistic value of $z_{0}$ = 3.0 mm, inspired by the fit result of model 1, to obtain a slightly worse but acceptable match with the experiments: $\sqrt{\left\langle\delta T^{2} \right\rangle}$ = 7 °C. Figure S10 shows the fit results. The qualitative trends are again reproduced: the higher temperatures in the narrow reactor (Figure S10b) compared to the wide reactor (Figure S10a) and lower temperatures in the middle of the catalyst bed (Figure S10c) compared to the top (Figure S10b).

Figure S10: Plots of the local temperature during the OCM process as a function of the heat generation (x axis) and oxygen conversion (y axis). The disks show the recorded excess catalyst temperature in the experiments, relative to the constant oven temperature under inert conditions. The background maps show model 2 (Equation (S12.15)), with fixed $z_{0}$ = 3.0 mm, and fitted values of $k$ = 0.93 W m^−1^ K^−1^ and $\alpha$ = 2.2 × 10^2^ W m^−2^ K^−1^, for experiments with **a**, 2$R$ = 7.1 mm measured in the top of the catalyst bed, **b**, 2$R$ = 4.1 mm measured in the top of the catalyst bed, **c**, 2$R$ = 4.1 mm measured 9.5 mm lower in the catalyst bed. All panels use a common color scale, which is identical to the scale used in Figure S8.

Figure S11: **a**, Profile of the heat generation rate $W(z)$ per unit of volume for selected experiments i and ii in Figure S10. $W(z)$ was the input for the fit model. The decay along $z$ is assumed exponential and determined by experimental values of $Q$ and $X_{O_{2}}$. The heat generation profile along $x$ (a cross section of the catalyst bed), is uniform by assumption. **b**, Profile of the temperature $T(z)$ according to the fit to model 2. The uniformity along $x$ is imposed by the model. Note the different temperature scale on the y axis compared to Figure S9b.

Figure S11 shows the corresponding heat generation and temperature profiles determined by model 2, for highlighted experiments i and ii in Figure S10. The flat temperature profile along the $\rho$ direction is imposed by the model. We can check whether the simplification of a flat radial profile is reasonable, using the fitted values of $k$ = 0.93 W m^−1^ K^−1^ and $\alpha$ = 2.2 × W m^−2^ K^−1^. Equations (S12.8)–(S12.11) from model 1 predict that the ratio $Y$ of the temperature difference between the center and the reactor wall over the temperature difference between the reactor wall and the oven is

$$Y=\frac{WR^{2}/4k}{WR/2\alpha}=\frac{R\alpha}{2k}$$

Equation (S12.18)

We calculate $Y$ = 0.42 for 2$R$ = 7.1 mm and $Y$ = 0.24 for 2$R$ = 4.1 mm. The higher value for the wider reactor makes sense: in reality, the heat diffusion in the radial direction is not infinitely fast, which will affect the thermal dynamics, depending on the reactor radius. Still, these relatively small values, especially for the narrow reactor, indicate that the approximation of a flat radial temperature profile, made for model 2, is reasonable for our analysis.

The model described here was derived for experiments with varying catalyst bed dimensions and measurement heights, to account for the $R$, $L$, and $z$ dependence of the recorded temperature. We assume a constant $k$ and $\alpha$ for these variations. Calculations described in Section 10 of the Supplementary Information yield a difference in effective thermal conductivity $\Delta\lambda_{\mathrm{eff}}$ < 1% between the different reactor tube diameters in our experiments, which is due to a slightly larger gas phase velocity for the same volumetric gas flow through a narrower reactor tube. This small change indicates that the assumption of a constant $k$ for the varying tube diameters is valid, keeping everything else constant. Because an increase in the catalyst grain size $d_{p}$ is expected to change the conductivity $k$ and the heat transfer coefficient $\alpha$, the corresponding measurements with larger $d_{p}$ were not considered in the derivation of the model. Instead, the model was derived for the experiments with $d_{p}$ = 300 µm as explained above, and subsequently fitted to the data with $d_{p}$ = 732 µm separately, to extract the corresponding values for $k$ and $\alpha$ as presented in the main text.

12.3. Discussion of the model

The modeling and fitting results provide intuitive explanations for the qualitative trends observed in the experiments. Irrespective of which model we use for the explanation, the exponentially decaying heat generation profile is central.

Model 1 has limited applicability to the system, because of its inconsistent description of heat diffusion along the $\rho$ and $z$ directions. Nevertheless, for completeness, the intuitive explanations according to model 1 would be as follows:

- The temperature at the top of the 2$R$ = 7.1 mm catalyst bed is lower than at the top of the 2$R$ = 4.1 mm bed because the measurement is done slightly below the very top (fitted $z_{0}$ = 2.8 mm). The heat generation rate peaks at $z_{0}$ = 0 mm and then decays more strongly with $z$ in the wider reactor than in the narrower reactor. As heat diffusion in the $z$ direction is neglected, this difference in heat-generation decay lengths translates directly into a difference in recorded temperature.
- The temperature in the middle of the 2$R$ = 4.1 mm bed is lower than at the top because the heat generation rate decays exponentially with $z$.

Model 2 seems to provide a more consistent description of the system. The intuitive explanations are as follows:

- The temperature at the top of the 2$R$ = 7.1 mm catalyst bed is lower than at the top of the 2$R$ = 4.1 mm bed because the heat generated can escape more effectively toward the quartz wool layer on top. Despite the higher peak of heat generation in the wider reactor (Figure S11a), the temperature peak is lower (Figure S11b). The heat flow across the interface from catalyst to quartz wool is $\pi R^{2}q_{z}(0)$ = 1.9 W for experiment i and $\pi R^{2}q_{z}(0)$ = 0.81 W for experiment ii. Compare this with the respective total heat generation rates of $Q$ = 5.6 W and $Q$ = 5.5 W, respectively.
- The temperature in the middle of the 2$R$ = 4.1 mm catalyst bed is lower than at the top because the heat generation rate decays exponentially with $z$.

13. Timescale of temperature dynamics

We describe a simple expression for the temperature dynamics over time, based on a total heat loss rate $k_{\mathrm{loss}}$ (s^−1^); the local catalyst temperature $T_{\mathrm{cat}}$ (K); the oven temperature under inert conditions $T_{\mathrm{oven}}$ (K); the total heat generation $Q$ (W); and an effective heat capacity $C_{\mathrm{eff}}$ (J K^−1^):

$$\frac{{dT}_{\mathrm{cat}}}{dt}=-k_{\mathrm{loss}}\left( T_{\mathrm{cat}}-T_{\mathrm{oven}} \right)+\frac{Q}{C_{\mathrm{eff}}}$$

Equation (S13.1)

The steady-state solution to Equation (S13.1) is

$$T_{\mathrm{cat}}=T_{\mathrm{oven}}+\frac{Q}{k_{\mathrm{loss}}C_{\mathrm{eff}}}$$

Equation (S13.2)

which is consistent with the roughly linear relation between the excess catalyst temperature as a function of the heat generation as shown in the main text. If we now imagine a change in heat generation $Q$ due to a change in the CH_4_/O_2_ ratio in the reactant feed, the steady-state catalyst temperatures $T_{\mathrm{cat},1}$ and $T_{cat,2}$ before and after the switch, respectively, are given by

$$T_{cat,1}=T_{\mathrm{oven}}+\frac{Q_{1}}{k_{\mathrm{loss}}C_{\mathrm{eff}}} T_{cat,2}=T_{\mathrm{oven}}+\frac{Q_{2}}{k_{\mathrm{loss}}C_{\mathrm{eff}}}$$

Equation (S13.3)

and the solution for temperature dynamics upon a change in $Q$ is

$$T_{\mathrm{cat}}\left( t \right)=T_{cat,2}+\left( T_{cat,1}-T_{cat,2} \right)e^{-k_{\mathrm{loss}}t}$$

Equation (S13.4)

Thus, upon a switch in the reactant flow, we expect an exponential response of the temperature over time, depending on the rate $k_{\mathrm{loss}}$.

Based on the heat loss rate $\gamma$ by transfer through the reactor wall, as defined in Equation (S12.13) in Section 12 of the Supplementary Information, we express the expected rate of temperature response $k_{loss,model}$ (unit s^−1^) upon an instantaneous switch in heat generation as a function of the heat transfer coefficient $\alpha$, reactor tube radius $R$, and heat capacity $C_{\text{p}}$:

$$k_{loss,model}=\frac{\gamma}{C_{\text{p}}}=\frac{2\alpha}{RC_{\text{p}}}$$

Equation (S13.5)

The description above essentially represents the lumped capacitance heat transfer model, in which spatial temperature variations are neglected. To check whether the result is reasonable for realistic conditions, we numerically calculated the response of a given heat generation and thus temperature profile upon removal of the heat generation (i.e., setting $W(z)$ = 0).

We use the $W(z)$ profile from experiment i in Figure S10a and Figure S11a (Fig. 7a in the main text), by discretization of equation S12.3 and using the corresponding best-fit values, catalyst bed dimensions, and reaction results. From that, we construct a $T(z)$ profile by iteration using Equation S12.14. After the iteration has converged into a stable temperature profile, we set $W(z)$ = 0 and calculate the resulting temperature profiles iteratively. Figure S12a shows the excess temperature relative to $T_{\mathrm{oven}}$ = 672 °C in the top of the catalyst bed ($z$ = 0 mm) as a function of the iteration steps (i.e., as a function of time). Figure S12b shows the excess temperature averaged over the whole catalyst bed as a function of iteration steps.

Figure S12: Numerically calculated temperature response upon removal of the heat generation $W(z)$, using the conditions and modelling results of experiment i in Figure S10. **a**, Excess temperature in the top of the catalyst bed as a function of iterations after removal of $W$. **b**, Average excess temperature over the catalyst bed as a function of iterations after removal of $W$. **c**, Calculated heat loss rate $k_{\text{num}}(z)$ (cyan) after removal of $W$ as a function of the $z$-coordinate in our numerical calculations. The calculated average heat loss rate $k_{\text{avg}}$ is shown in purple as a reference.

For both the temperature in the top of the catalyst bed (Figure S12a) and the average temperature over the whole catalyst bed (Figure S12b), we observe an exponential decrease in temperature after the removal of $W$, which is consistent with our expectations explained above. We extract the heat loss rate $k_{\text{num}}(z)$ from the calculated decay in temperature by fitting the data to an exponential decay in the form of Equation S13.4. For the average temperature response of Figure S12b, we find $k_{\text{avg}}$ = 0.043 s^−1^, which is consistent with the calculation of $k_{\text{loss,model}}$ = 0.043 s^−1^ from Equation S13.5 using the same conditions and modelling results.

Figure S12c shows the extracted best-fit values of $k_{\text{num}}(z)$ over the catalyst bed (cyan). We see that indeed, the temperature response rate is not constant over the whole catalyst bed. However, the deviation from $k_{\text{avg}}$ is within ~ 16% over the whole catalyst bed, indicating that the transient LT experiments and analysis thereof can provide qualitative insight into the temperature dynamics.

14. Supplementary References

1.	Brites, C. D. S., Balabhadra, S. & Carlos, L. D. Lanthanide‐Based Thermometers: At the Cutting‐Edge of Luminescence Thermometry. *Adv. Opt. Mater.* **7**, 1801239 (2019).

2.	Dramićanin, M. D. Trends in luminescence thermometry. *J. Appl. Phys.* **128**, 040902 (2020).

3.	Goss, L. P., Smith, A. A. & Post, M. E. Surface thermometry by laser‐induced fluorescence. *Rev. Sci. Instrum.* **60**, 3702–3706 (1989).

4.	Allison, S. W. & Gillies, G. T. Remote thermometry with thermographic phosphors: Instrumentation and applications. *Rev. Sci. Instrum.* **68**, 2615–2650 (1997).

5.	Chambers, M. D. & Clarke, D. R. Doped Oxides for High-Temperature Luminescence and Lifetime Thermometry. *Annu. Rev. Mater. Res.* **39**, 325–359 (2009).

6.	Brübach, J., Pflitsch, C., Dreizler, A. & Atakan, B. On surface temperature measurements with thermographic phosphors: A review. *Prog. Energy Combust. Sci.* **39**, 37–60 (2013).

7.	Hertle, E., Chepyga, L., Batentschuk, M. & Zigan, L. Influence of codoping on the luminescence properties of YAG:Dy for high temperature phosphor thermometry. *J. Lumin.* **182**, 200–207 (2017).

8.	Geitenbeek, R. G. *et al.* *In situ* luminescence thermometry to locally measure temperature gradients during catalytic reactions. *ACS Catal.* **8**, 2397–2401 (2018).

9.	Hartman, T., Geitenbeek, R. G., Whiting, G. T. & Weckhuysen, B. M. *Operando* monitoring of temperature and active species at the single catalyst particle level. *Nat. Catal.* **2**, 986–996 (2019).

10.	Back, M. *et al.* Boltzmann Thermometry in Cr^3+^‐Doped Ga_2_O_3_ Polymorphs: The Structure Matters! *Adv. Opt. Mater.* **9**, 2100033 (2021).

11.	Terlingen, B. J. P. *et al.* Bifunctional Europium for *Operando* Catalyst Thermometry in an Exothermic Chemical Reaction. *Angew. Chem. Int. Ed.* **61**, e202211991 (2022).

12.	Ye, Z., Bommidi, D. K. & Pickel, A. D. Dual‐Mode *Operando* Raman Spectroscopy and Upconversion Thermometry for Probing Thermal Contributions to Plasmonic Photocatalysis. *Adv. Opt. Mater.* **11**, 2300824 (2023).

13.	Farpón, M. G. *et al.* Multifunctional materials for catalyst-specific heating and thermometry in tandem catalysis. *J. Mater. Chem. A* **11**, 19854–19859 (2023).

14.	Jacobs, T. S. *et al.* Mapping Temperature Heterogeneities during Catalytic CO_2_ Methanation with *Operando* Luminescence Thermometry. *ACS Nano* **17**, 20053–20061 (2023).

15.	Tian, Y. *et al.* Spatiotemporal Heterogeneity of Temperature and Catalytic Activation within Individual Catalyst Particles. *J. Am. Chem. Soc.* **146**, 4958–4972 (2024).

16.	Dorresteijn, J. M. *et al.* *In-Situ* Thermometry Reveals Fragmentation Behavior Based on Local Temperature in α-Olefin Polymerization Catalysts. *J. Am. Chem. Soc.* **147**, 5642–5648 (2025).

17.	Vogel, R. *et al.* *Operando* Luminescence Thermometry for Hydrocarbon Conversion Catalysis: Dealing with Dynamic Changes in Catalyst Optical Properties. *ACS Appl. Mater. Interfaces* **17**, 21215–21222 (2025).

18.	Perera, S. M. H. D., Harrington, B., Fadhul, A., Pickel, A. D. & Porosoff, M. D. Leveraging and understanding exotherms in tandem catalysts with in situ luminescence thermometry. *EES Catal.* **4**, 333–340 (2026).

19.	Groefsema, D. W., Rabouw, F. T., Boele, M., van Bavel, A. P. & Weckhuysen, B. M. *Operando* Luminescence Thermometry of a Solid Catalyst in a Reactor during a High‐Temperature Chemical Process. *Chemistry–Methods* **5**, e202400044 (2025).

20.	Olivos-Suarez, A. I. *et al.* Strategies for the direct catalytic valorization of methane using heterogeneous catalysis: challenges and Opportunities. *ACS Catal.* **6**, 2965–2981 (2016).

21.	Lim, S. *et al.* Low-temperature oxidative coupling of methane using alkaline earth metal oxide-supported perovskites. *Catal. Today* **352**, 127–133 (2020).

22.	Kim, M. *et al.* Reaction engineering of oxidative coupling of methane. *Chem. Eng. Res. Des.* **172**, 84–98 (2021).

23.	Ortiz-Bravo, C. A., Chagas, C. A. & Toniolo, F. S. Oxidative coupling of methane (OCM): An overview of the challenges and opportunities for developing new technologies. *J. Nat. Gas Sci. Eng.* **96**, 104254 (2021).

24.	Alturkistani, S., Wang, H., Gautam, R. & Sarathy, S. M. Importance of Process Variables and Their Optimization for Oxidative Coupling of Methane (OCM). *ACS Omega* **8**, 21223–21236 (2023).

25.	Godini, H. R. & Bhasin, M. M. Oxidative Coupling of Methane: A Review Study on the Catalytic Performance. *Molecules* **29**, 4649 (2024).

26.	Ross, D., Gaitan, M. & Locascio, L. E. Temperature Measurement in Microfluidic Systems Using a Temperature-Dependent Fluorescent Dye. *Anal. Chem.* **73**, 4117–4123 (2001).

27.	Benninger, R. K. P. *et al.* Quantitative 3D Mapping of Fluidic Temperatures within Microchannel Networks Using Fluorescence Lifetime Imaging. *Anal. Chem.* **78**, 2272–2278 (2006).

28.	Li, S., Zhang, K., Yang, J.-M., Lin, L. & Yang, H. Single Quantum Dots as Local Temperature Markers. *Nano Lett.* **7**, 3102–3105 (2007).

29.	Omrane, A., Petersson, P., Aldén, M. & Linne, M. A. Simultaneous 2D flow velocity and gas temperature measurements using thermographic phosphors. *Appl. Phys. B* **92**, 99–102 (2008).

30.	Särner, G., Richter, M. & Aldén, M. Two-dimensional thermometry using temperature-induced line shifts of ZnO:Zn and ZnO:Ga fluorescence. *Opt. Lett.* **33**, 1327 (2008).

31.	Saïdi, E. *et al.* Tuning Temperature and Size of Hot Spots and Hot‐Spot Arrays. *Small* **7**, 259–264 (2011).

32.	Bennet, M. A. *et al.* Optically trapped microsensors for microfluidic temperature measurement by fluorescence lifetime imaging microscopy. *Lab Chip* **11**, 3821 (2011).

33.	Shinde, S. L. & Nanda, K. K. Wide‐Range Temperature Sensing using Highly Sensitive Green‐Luminescent ZnO and PMMA‐ZnO Film as a Non‐Contact Optical Probe. *Angew. Chem. Int. Ed.* **52**, 11325–11328 (2013).

34.	Wu, J. *et al.* Mapping three-dimensional temperature in microfluidic chip. *Sci. Rep.* **3**, 3321 (2013).

35.	Gomez, G. E. *et al.* Photoluminescence, Unconventional‐Range Temperature Sensing, and Efficient Catalytic Activities of Lanthanide Metal–Organic Frameworks. *Eur. J. Inorg. Chem.* **2016**, 1577–1588 (2016).

36.	Abed, W. M., Domingues, A. F., Poole, R. J. & Dennis, D. J. C. Heat transfer enhancement in a cross-slot micro-geometry. *Int. J. Therm. Sci.* **121**, 249–265 (2017).

37.	Runowski, M. *et al.* Multifunctional Optical Sensors for Nanomanometry and Nanothermometry: High-Pressure and High-Temperature Upconversion Luminescence of Lanthanide-Doped Phosphates—LaPO_4_/YPO_4_:Yb^3+^–Tm^3+^. *ACS Appl. Mater. Interfaces* **10**, 17269–17279 (2018).

38.	Geitenbeek, R. G. *et al.* Luminescence thermometry for *in situ* temperature measurements in microfluidic devices. *Lab Chip* **19**, 1236–1246 (2019).

39.	Pfaff, S., Karlsson, H., Nada, F. A., Lundgren, E. & Zetterberg, J. Temperature characterization of an *operando* flow reactor for heterogeneous catalysis. *J. Phys. D. Appl. Phys.* **52**, 324003 (2019).

40.	van Ravenhorst, I. K. *et al.* *In Situ* Local Temperature Mapping in Microscopy Nano‐Reactors with Luminescence Thermometry. *ChemCatChem* **11**, 5505–5512 (2019).

41.	Lin, X. *et al.* Measurement of Temperature Distribution at the Nanoscale with Luminescent Probes Based on Lanthanide Nanoparticles and Quantum Dots. *ACS Appl. Mater. Interfaces* **12**, 52393–52401 (2020).

42.	van Swieten, T. P. *et al.* Mapping Elevated Temperatures with a Micrometer Resolution Using the Luminescence of Chemically Stable Upconversion Nanoparticles. *ACS Appl. Nano Mater.* **4**, 4208–4215 (2021).

43.	Piotrowski, W. M. *et al.* Positive luminescence thermal coefficient of Mn^2+^ ions for highly sensitive luminescence thermometry. *Chem. Eng. J.* **464**, 142492 (2023).

44.	Xuan, G. *et al.* Multi-point temperature measurements in packed beds using phosphor thermometry and ray tracing simulations. *Particuology* **85**, 77–88 (2024).

45.	Wang, Y., Zhang, Q., Yang, C. & Xia, Z. Ratiometric Fluorescence Optical Fiber Enabling *Operando* Temperature Monitoring in Pouch‐Type Battery. *Adv. Mater.* **36**, 2401057 (2024).

46.	Ma, Y. *et al.* Luminescence Thermometry via Multiparameter Sensing in YV_1– x_P_x_O_4_:Eu^3+^,Er^3+^. *J. Am. Chem. Soc.* **147**, 12925–12936 (2025).

47.	Birch, K. P. & Downs, M. J. Correction to the Updated Edlén Equation for the Refractive Index of Air. *Metrologia* **31**, 315–316 (1994).

48.	Linstrom, P. J. & Mallard, W. G. NIST Chemistry WebBook. *NIST Standard Reference Database Number 69*
https://webbook.nist.gov/chemistry
(accessed 14-04-2023).

49.	Yagi, S. & Kunii, D. Studies on effective thermal conductivities in packed beds. *AIChE J.* **3**, 373–381 (1957).

50.	Kunii, D. & Smith, J. M. Heat transfer characteristics of porous rocks. *AIChE J.* **6**, 71–78 (1960).

51.	Zehner, P. & Schlünder, E. U. Einfluß der wärmestrahlung und des druckes auf den wärmetransport in nicht durchströmten schüttungen. *Chem. Ing. Tech.* **44**, 1303–1308 (1972).

52.	Froment, G. F., Bischoff, K. B. & De Wilde, J. *Chemical Reactor Analysis and Design*. (Wiley, 2011).

53.	Klein, P. H. & Croft, W. J. Thermal Conductivity, diffusivity, and expansion of Y_2_O_3_, Y_3_Al_5_O_12_, and LaF_3_ in the range 77–300 K. *J. Appl. Phys.* **38**, 1603–1607 (1967).

54.	Yin, D. *et al.* Fabrication of highly transparent Y_2_O_3_ ceramics with CaO as sintering aid. *Materials* **14**, 444 (2021).

55.	American Elements. *Y2O3 product page*
https://www.americanelements.com/yttrium-oxide-1314-36-9
(accessed 01-10-2023).

56.	CoorsTek. *Y_2_O_3_ product page*
https://www.coorstek.com/en/materials/yttria (accessed 01-10-2023).
